# Supplementary material for: NeuroMotion smartphone application for remote General Movements Assessment: a feasibility study in Nepal
Source: BMJ Open. 2024 Mar 1;14(3):e080063. doi: 10.1136/bmjopen-2023-080063 (PMC10910581; doi:10.1136/bmjopen-2023-080063)
Supplement: Supplementary data [file bmjopen-2023-080063supp001.pdf]

Film #

Assessed by:____

Date:____

Quality	Approved		Not approved	Comments
	Excellent	Good	Indistinct	
Picture quality				
Sharpness				
Camera is held in correct position				
Camera is held in a steady position				
Baby setting				
Clothing				
Background/blanket				
Supine position				
Surrounding influences				
Baby´s mood				
Overall judgement:				
