# Supplementary material for: NeuroMotion smartphone application for remote General Movements Assessment: a feasibility study in Nepal
Source: BMJ Open. 2024 Mar 1;14(3):e080063. doi: 10.1136/bmjopen-2023-080063 (PMC10910581; doi:10.1136/bmjopen-2023-080063)
Supplement: Supplementary data [file bmjopen-2023-080063supp002.pdf]

		Picture quality			Baby setting					Overall judgement	Fidgety Movements
Film number	Case number	Sharpness	Camera held in correct pos.	Camera held steady	Clothing	Background/ blanket	Supine position	Surrounding influences	Baby's mood		
1	1	G	E	G	E	E	E	E	E	Approved	F-
2	1	G	I	G	E	E	E	E	E	Approved	F-
3	1	G	G	G	E	E	E	E	E	Approved	F-
4	2	E	I	I	E	G	E	E	E	Approved	F+
5	2	E	I	I	E	E	E	E	E	Rejected	?
6	2	E	I	I	E	G	E	E	E	Approved	F+
7	3	E	E	I	E	G	E	E	E	Approved	F+
8	4	E	E	G	E	G	E	E	E	Approved	F+
9	4	E	E	G	E	G	E	E	E	Approved	F+
10	4	E	E	G	E	G	E	G	E	Approved	F+
11	4	E	E	G	E	G	G	I	E	Rejected	F+
12	4	E	I	I	E	G	G	I	E	Rejected	?
13	4	E	E	E	E	G	E	G	E	Approved	F+
14	5	E	E	E	G	E	E	E	E	Approved	F+
15	5	E	E	E	G	I	E	E	E	Approved	F+
16	5	E	G	E	E	G	E	E	E	Approved	F+
17	5	E	E	E	E	E	E	E	E	Approved	F+
18	5	E	E	E	E	E	E	E	E	Approved	F+
19	6	E	E	I	E	E	E	E	E	Approved	F+
20	6	E	E	G	E	E	E	E	E	Approved	F+
21	7	E	E	G	E	I	E	I	G	Approved	F+
22	7	E	E	I	G	I	E	E	E	Approved	F+
23	8	E	I	I	G	G	E	I	E	Rejected	F+
24	9	G	G	G	E	I	E	G	E	Approved	F+
25	10	E	G	G	E	G	E	G	E	Approved	F-
26	10	G	E	I	E	I	E	I	E	Approved	F-
27	10	E	G	I	E	G	E	G	E	Approved	F-
28	11	E	I	I	I	I	E	E	E	Rejected	?
29	11	E	I	I	E	E	E	I	E	Rejected	F+
30	12	I	E	G	I	G	E	E	E	Rejected	?
31	12	I	G	G	I	I	G	E	E	Rejected	F+
32	12	E	G	E	G	E	E	E	E	Approved	F+
33	12	E	G	G	I	I	E	I	E	Approved	F+
34	13	E	G	G	E	E	E	E	E	Approved	F+
35	13	E	E	G	E	E	E	E	E	Approved	F+
36	14	E	I	I	E	I	E	I	E	Rejected	F-(?)
37	15	E	G	G	E	E	E	E	E	Approved	F+

38	16	E	I	I	I	I	E	I	E	Rejected	?
39	16	E	E	E	G	E	E	E	E	Approved	F+
40	16	E	E	E	G	E	E	E	E	Approved	F+
41	17	E	E	E	E	E	E	E	E	Approved	F+
42	18	E	E	E	E	E	E	E	E	Approved	F+
43	18	E	E	E	E	E	E	G	E	Approved	F+
44	19	E	G	E	E	E	E	E	E	Approved	F+
45	19	E	I	G	E	G	E	E	E	Approved	F+
46	20	E	I	I	E	I	E	E	E	Rejected	F+
Summary of results	Excellent	39	23	13	34	20	43	31	45		
	Good	5	11	18	7	15	3	6	1		
	Indistinct	2	12	15	5	11	0	9	0		
Films number 11, 23, 29 and 31 were rejected for technical quality, but Fidgety Movements were clearly observed.											
Case number 14 only sent one film that was rejected due to poor technical quality, but Fidgety Movements were not observed.											
