# Supplementary material for: NeuroMotion smartphone application for remote General Movements Assessment: a feasibility study in Nepal
Source: BMJ Open. 2024 Mar 1;14(3):e080063. doi: 10.1136/bmjopen-2023-080063 (PMC10910581; doi:10.1136/bmjopen-2023-080063)
Supplement: Author's manuscript [file bmjopen-2023-080063.draft_revisions.pdf]

BMJ Open

BMJ Open is committed to open peer review. As part of this commitment we make the peer review history of every article we publish publicly available.

When an article is published we post the peer reviewers' comments and the authors' responses online. We also post the versions of the paper that were used during peer review. These are the versions that the peer review comments apply to.

The versions of the paper that follow are the versions that were submitted during the peer review process. They are not the versions of record or the final published versions. They should not be cited or distributed as the published version of this manuscript.

BMJ Open is an open access journal and the full, final, typeset and author-corrected version of record of the manuscript is available on our site with no access controls, subscription charges or pay-per-view fees (http://bmjopen.bmj.com).

If you have any questions on BMJ Open's open peer review process please email info.bmjopen@bmj.com

BMJ Open

NeuroMotion™ Smartphone Application for Remote General Movements Assessment: a Feasibility Study in Nepal

Journal:	*BMJ Open*
Manuscript ID	bmjopen-2023-080063
Article Type:	Original research
Date Submitted by the Author:	22-Sep-2023
Complete List of Authors:	Kukka, Antti; Uppsala University, Department of Women's and Children's Health; Region Gävleborg, Department of Pediatrics<br>Sundelin, Heléne; Linköping University, Department of Biomedical and Clinical Sciences; Karolinska Institutet, Department of Women's and Children's Health<br>Basnet, Omkar; Golden Community<br>Paudel, Prajwal; Paropakar Maternity and Women's Hospital<br>Subedi, Kalpana; Paropakar Maternity and Women's Hospital<br>Svensson, Katarina; Linköping University, Division of Children's and Women's Health, Department of Biomedical and Clinical Sciences<br>brown, nick; Uppsala Universitet, Department of Women's and Children's Health; Region Gävleborg, Department of Pediatrics<br>Litorp, Helena; Uppsala University, Department of Women's and Children's Health; Karolinska Institutet, Department of Global Public Health<br>Gurung, Rejina; Uppsala University, Department of Women's and Children's Health; Golden Community<br>Bhattarai, Pratiksha; Golden Community<br>Wrammert, Johan; Uppsala University, Department of Women's and Children's Health<br>Ashish, KC; Uppsala University, Department of Women's and Children's Health; University of Gothenburg, School of Public Health and Community Medicine
Keywords:	Developmental neurology & neurodisability < PAEDIATRICS, Feasibility Studies, Nepal, Follow-Up Studies, NEONATOLOGY

SCHOLARONE™
Manuscripts

I, the Submitting Author has the right to grant and does grant on behalf of all authors of the Work (as defined in the below author licence), an exclusive licence and/or a non-exclusive licence for contributions from authors who are: i) UK Crown employees; ii) where BMJ has agreed a CC-BY licence shall apply, and/or iii) in accordance with the terms applicable for US Federal Government officers or employees acting as part of their official duties; on a worldwide, perpetual, irrevocable, royalty-free basis to BMJ Publishing Group Ltd ("BMJ") its licensees and where the relevant Journal is co-owned by BMJ to the co-owners of the Journal, to publish the Work in this journal and any other BMJ products and to exploit all rights, as set out in our licence.

The Submitting Author accepts and understands that any supply made under these terms is made by BMJ to the Submitting Author unless you are acting as an employee on behalf of your employer or a postgraduate student of an affiliated institution which is paying any applicable article publishing charge ("APC") for Open Access articles. Where the Submitting Author wishes to make the Work available on an Open Access basis (and intends to pay the relevant APC), the terms of reuse of such Open Access shall be governed by a Creative Commons licence – details of these licences and which Creative Commons licence will apply to this Work are set out in our licence referred to above.

Other than as permitted in any relevant BMJ Author's Self Archiving Policies, I confirm this Work has not been accepted for publication elsewhere, is not being considered for publication elsewhere and does not duplicate material already published. I confirm all authors consent to publication of this Work and authorise the granting of this licence.

For peer review only

NeuroMotion™ Smartphone Application for Remote General Movements Assessment: a Feasibility Study in Nepal

Author list:

Antti J. Kukka[1,2], Heléne E.K. Sundelin[3,4], Omkar Basnet[5], Prajwal Paudel[6], Kalpana Upadhyaya Subedi[6], Katarina Svensson[3,7], Nick J.W. Brown[1,2], Helena Litorp[1,8], Rejina Gurung[1,5], Pratiksha Bhattarai[5], Johan Wrammert[1]and Ashish KC[1,9]

Affiliations:

1. Department of Women's and Children's Health, Uppsala University, Sweden
2. Department of Pediatrics, Gävle Regional Hospital, Region Gävleborg, Sweden
3. Division of Children´s and Women´s Health, Department of Biomedical and Clinical Sciences, Linköping University, Linköping, Sweden.
4. Neuropediatric Unit, Department of Women's and Children's Health, Karolinska University Hospital, Karolinska Institutet, Stockholm, Sweden
5. Golden Community, Lalitpur, Nepal
6. Paropakar Maternity and Women's Hospital, Kathmandu, Nepal
7. Crown Princess Victoria's Children's and Youth Hospital, University Hospital, Linkoping, Sweden
8. Department of Global Public Health, Karolinska Institutet, Stockholm, Sweden
9. School of Public Health and Community Medicine, University of Gothenburg, Gothenburg, Sweden

Corresponding author: Antti J Kukka, Department of Women's and Children's Health, Uppsala University, SE-751 85, Uppsala, Sweden, https://www.katalog.uu.se/profile/?id=N19-2319 Email address: antti.kukka@uu.se

Abstract:

OBJECTIVES: To evaluate the feasibility of using the NeuroMotion™ smartphone application for remote General Movements Assessment for screening infants for cerebral palsy in Kathmandu, Nepal.

DESIGN: Pilot study.

SETTING: Single tertiary delivery hospital with a sick newborn care unit and a neonatal intensive care unit.

PARTICIPANTS: Thirty-one term-born infants at risk of cerebral palsy due to birth asphyxia (Apgar ≤5 at 5min, positive pressure ventilation ≥5 min) or clinical seizures were recruited for follow-up. Participants were excluded in case of residence outside of Kathmandu Valley or need of transfer to another hospital.

INTERVENTIONS: Parents filmed their children at home using the app at 3 months' age and the videos were assessed for technical quality using a standardized form and for fidgety movements

For peer review only

by Prechtl's General Movements Assessment. Usability of the app was evaluated by a parental survey, and the results were compared with findings from previous studies in high-income countries.

PRIMARY OUTCOMES: Reach, quality and usability of the intervention.

RESULTS: Twenty families sent 46 videos, of which 35 had acceptable technical quality. Sixteen children had at least one video with acceptable technical quality, and three of them lacked fidgety movements. The level of agreement between assessors was acceptable (Krippendorf alpha 0.781). Parental answers to the usability survey (n=25) were comparable with results from high-income countries.

CONCLUSION: Engaging parents in the screening of cerebral palsy with the help of a smartphone-aided remote General Movements Assessment is possible in the urban area of a South Asian lower middle-income country. Scale-up of the screening intervention is likely to require substantial resources.

Article summary:

Strengths and limitations

- Usability of the app was assessed by both parents who succeeded and failed in filming their child.
- The planned sample size was not reached.
- Results of the study might not be generalizable to rural areas of Nepal.
- Feasibility of the remote follow-up for the healthcare providers was not assessed.

Funding: The project was funded by grants from Promobilia stiftelse, Linnea and Josef Carlssons stiftelse, Folke Bernadotte Stiftelse and Födelsefonden. Heléne Sundelin is funded by Region Stockholm, clinical postdoctoral appointment, 2019-1138 and a private donation through the Knut and Alice Wallenberg Foundation.

Competing interests:
KS and HEKS developed the app NeuroMotion™ with financial support from a private donation through Knut och Alice Wallenbergs Stiftelse. For the purpose of further research and later implementation of NeuroMotion™, the company NeuroMotion AB was founded and is the owner of the app. NeuroMotion AB is owned by KS and HEKS and has the purpose of research and to be nonprofitable.

Data sharing statement:

Data available on request from the authors: The data that support the findings of this study are available from the corresponding author upon reasonable request.

Word count: 2960
Key words: cerebral palsy, general movements assessment, neonatal follow-up, low- and middle-income countries, pilot projects

For peer review only - http://bmjopen.bmj.com/site/about/guidelines.xhtml

Background

All children have the right to not only survive birth but to also thrive and reach their full developmental potential.[1] Cerebral palsy (CP) is the most common motor disability of childhood, and international guidelines recommend early intervention for all children at high risk of CP.[2] A number of on-going trials are evaluating ways to adapt early intervention to low- and middle-income countries (LMICs),[3–5] which shoulder the vast majority of children affected by CP globally.[6] Delay in accessing rehabilitation is common due to poor availability and lack of targeted screening programs for high-risk children.[7,8]

Affordable and accurate tools are therefore needed to identify children at high risk of CP in LMICs. General Movements Assessment (GMA) is an inexpensive, non-invasive method for recognizing these children based on observing their spontaneous movements.[9] It has been shown to have excellent validity at 3 months' age, and its use in screening high-risk children is now globally endorsed.[10,11] However, a lack of providers trained in the methodology has thus far limited its implementation,[12] and telemedicine is one way of overcoming this barrier.[13,14] Lockdowns imposed during the recent Covid-19 pandemic have increased the urgency of finding alternatives to traditional face-to-face checkups.

Previous studies in high-income countries (HICs) have shown that remote GMA using smartphone applications (apps) is feasible.[15–17] Automatized movement analysis using machine learning holds further promise for wider availability.[18] However, to our knowledge, no previous study has utilized smartphone apps for remote GMA in LMIC settings, where the disease burden is the greatest despite rapidly increasing access to technology. As the validity of remote GMA outside of high-income countries is unclear, we conducted a pilot study to evaluate the feasibility of using smartphone-aided remote GMA for screening infants at risk of CP in Kathmandu, the capital of Nepal, a lower middle-income country.

Methods

A combination of the STROBE checklist for cohort studies[19] and CONSORT statement for pilot and feasibility trials[20] was used for reporting the study following Guidelines for reporting non-randomised pilot and feasibility studies.[21]

Study design and setting

This is a single-centre feasibility study conducted at the Paropakar Maternity and Women's Hospital, Kathmandu, Nepal. Recruitment of infants ran from 1st October 2021 to 7th January 2022 just before the onset of the third peak of the Covid-19 pandemic, and the last response to the usability survey was registered on 2nd March 2023. Paropakar is a public tertiary hospital acting as the referral hospital for the surrounding Bagmati Province. Infants admitted to the hospital's Neonatal Intensive Care Unit/Sick Newborn Care Unit (NICU/SNCU) are offered post-discharge follow-up at the paediatric outpatient department up to 14 years of age, but few families choose to

For peer review only - http://bmjopen.bmj.com/site/about/guidelines.xhtml

participate. Nationally, only 70% of infants receive a post-natal check-up in Nepal[22,] and targeted developmental clinics for high-risk children are generally lacking. The ownership of smartphones in Bagmati Province is 75 and 84% among women and men, respectively.[22]

Participants

Term born neonates (≥ 37 completed gestational weeks according to last menstrual period) at risk of CP due to birth asphyxia or seizures were recruited for follow-up. The included infants had to survive to discharge and fulfil at least one of the following criteria: 1) Apgar score ≤ 5 at 5 minutes; 2) bag-and-mask ventilation ≥ 5 minutes after delivery; or 3) diagnosis of clinical seizures at the NICU/SNCU.

Newborn infants who were transferred to other hospitals due to a lack of ventilators or the need for surgery and those whose parents lived outside of the Kathmandu Valley with a travel time to the hospital above one hour were excluded.

Hospital guidelines mandated that all eligible infants be admitted to the NICU/SNCU, which was confirmed by a chart review of 3 preceding months. Paediatricians working at the wards identified eligible infants and informed a research assistant in charge of recruitment. Background demographic data and delivery details were collected by a short parental interview and patient chart review, respectively. All participating parents were contacted by a phone call when their child was six weeks old to enquire about the health of the child and to give parents a possibility to ask further questions about the study.

Sample size

A sample of 40 neonates was planned for based on a sample size calculation of approximately 400 infants needed to reliably show sensitivity and specificity of the GMA screening tool for later CP diagnosis in a full-scale study. The sample of 40 was deemed large enough for testing the feasibility of recruitment, evaluating the rate of successful filming and gathering parental perceptions of the app usability. Based on our previous experience from the hospital, approximately 2% of deliveries were expected to meet the inclusion criteria.[23]

Intervention

The NeuroMotion™ app was translated from Swedish to Nepali and English and adapted to the local context by adding pictures and the possibility of storing videos on the phone for later submission if the internet was unavailable at the time of filming. A trained research assistant guided the parents in installing the app on their smartphones. Due to delay of the app modification, parents of the first 24 infants were instructed to do the installation at home, while nine families received the app before discharge from the hospital, and two parents without smartphones were invited to come to the hospital to have their child filmed by a research assistant.

All parents were instructed to film two videos of their child during the fidgety movements (FM) period 12-16 weeks post-term age.[9] The app automatically sent notifications to the parents when

For peer review only

For peer review only - http://bmjopen.bmj.com/site/about/guidelines.xhtml

their child was 12 weeks post-term age. The first notification was sent 3 days before scheduled filming, the second on the day of the filming and the third 3 days after the scheduled date if no film had been sent in by then. The research team contacted the parents by phone if no film had been uploaded after the third notification. A second film was requested by a notification in the app two days after the first film had been sent or by phone after the film analysis, whichever came first.

The filming took place at home or at the hospital by positioning the child in a supine position when awake and satisfied with arms and legs visible in the screen according to the GMA guidelines.[9] The duration of the films was 2 to 3 minutes each, as in previous studies.[16,17] All videos were analysed according to GMA by three independent certified evaluators blinded to the medical history of the patient.[9] The results were recorded as present (continuous, intermittent or abnormal) or absent (sporadic or absent) FMs. The assessments were discussed in meetings held every second week. When more than one film was available for a child, the best result category was recorded. A single evaluator (HS) assessed the technical quality of the films based on a standardized formula (Supplementary material).

The final consensus agreement was reported to the researchers in Nepal, who in turn informed the families via phone. In case of poor technical quality or absent FMs, one further recording was requested. Babies with consistently absent FMs were referred for an evaluation by a paediatrician at the Paropakar Maternity and Women's Hospital and were offered a referral for an early intervention programme at the Self-help Group for Cerebral Palsy, Lalitpur, after parental consent. The costs of the rehabilitation were covered by the research team.

After receiving feedback about their child's GMA, parents were interviewed by phone about usability and perception of the app by applying a multiple-choice questionnaire in Nepali. The survey consisted of 19 questions with answers on a five-point Likert scale with an option for free text answer. The questions were based on a survey developed by Kwong et al. for evaluation of the BabyMoves app in Australia.[24] Similar questions have also been used in studies in Europe.[16,17]

Implementation outcomes and statistical analyses

Feasibility was defined as the extent to which smartphone-aided remote screening could be successfully carried out within our setting.[25] The following implementation outcomes were measured quantitatively:

a) *The reach* of the screening program was evaluated by the proportion of participating children in each stage of the follow-up. Background factors between children and parents who successfully returned at least one scorable film were compared with those who did not by using an independent samples t-test for normally distributed continuous variables and Pearson's chi-square test or Fischer's exact test for categorical variables depending on cell count. $P<0.05$ was considered statistically significant. Crude odds ratios with 95 % confidence intervals were counted for the categorical variables.

For peer review only - http://bmjopen.bmj.com/site/about/guidelines.xhtml

b) *The quality* of the videos produced was evaluated by a standardized GMA Trust questionnaire, and the quality of the assessment was evaluated by calculating interevaluator agreement with Krippendorf alpha for videos with acceptable technical quality.[26]

c) Parental perceptions of the *usability* of the app were evaluated by a multiple-choice questionnaire, and the results were compared with previous studies from high-income settings using stacked bar graphs.[16,17,24]

Qualitative evaluation of the acceptability of the intervention will be reported separately (submitted manuscript).

Patient and public involvement

No patients were involved in the design of this study.

Ethics

Oral and written information was provided to at least one of the parents, and written consent was collected prior to enrolment. The research assistant was hired for the study and not part of the clinical care team. Ethical permit for the study was granted by the Institutional Review Committee of the Paropakar Maternity and Women's Hospital (61/1978) and for data analysis by the Swedish Ethical Review Authority (2020-07168).

Results

Reach and Quality

The rate of birth asphyxia was lower than expected during the study period, and more families were ineligible due to residence outside of the study area. Furthermore, during the first half of the study, mortality at the NICU/SNCU was higher than expected, and more infants were transferred to other hospitals. Therefore, only 34 eligible infants were identified, and 31 of them were enrolled despite extension of the recruitment period by a month (Figure 1). Male predominance was notable, with only six female infants recruited into the study.

Altogether, 46 videos were received from 20 families. All films were made between 12 and 16 weeks of post-term age and thus suitable for GMA analysis. The technical quality of the videos was acceptable in 35 out of 46 cases (Supplementary Table 1), and among films of 16 children, at least one was of good technical quality. The most common quality issues leading to the rejection of videos were inability to hold the camera still (15/46), wrong position of the camera for filming (12/46) and a background that was too busy (11/46). The level of agreement between the evaluators was acceptable (Krippendorf alpha 0.781 that all three assessors agreed).

There were no statistically significant differences in the characteristics between children whose parents succeeded in returning at least one scorable film versus those who did not (Table 1). Three children had consistently absent FMs. One family moved abroad and could not be reached for

For peer review only - http://bmjopen.bmj.com/site/about/guidelines.xhtml

follow-up. Two children were evaluated by a paediatrician (PP) and recommended early intervention, but their parents decided against joining the programme.

Usability

A response to the usability survey was provided by parents of 25 participating children including 18 parents who had succeeded in filming their child and seven who had attempted to use the app but not succeeded in sending a video. The answers were comparable to previous studies from high-income settings (Figure 2). Most of the parents found that the app was easy to use, and instructions were easy to follow. The screen filter outlining the contours of a child helped parents position the camera, and sending the videos was easy in most cases. More than half of the participants experienced worry about their child's development when using the app, and most felt that using the app increased their awareness of their child's development. A face-to-face assessment would have been preferable to filming for 8/25 respondents.

Discussion

This study examined the feasibility of implementing smartphone-aided remote GMA for screening infants at high risk of CP in Nepal. We found that just under half of eligible parents (16/34) were able to return at least one film of acceptable technical quality. Overall, 35 out of 46 films were of acceptable technical quality. Parental perceptions of the follow-up were positive, but parents to children with absent fidgety movements were not willing to join the offered early intervention program.

To our knowledge, this is the first time that a smartphone app has been used for assessing GMA outside high-income settings. Our group was more likely to consent to participate (31/34, 91%) than that reported in an Australian cohort of preterm infants (227/273, 83%).[24] However, the proportion successfully filming was lower in Nepal (16/31, 52%) than in Australia (158/226, 70%),[24] Belgium, Denmark and Norway (69/86, 80%),[24] or Sweden (36/37, 97%).[17] This was mostly due to the technical quality of films being lower than in the previous studies that all reported acceptable quality in more than 95% of the videos. This contrasts with the findings of the usability survey, where the vast majority of the Nepalese parents reported that they found using the app easy and instructions easy to follow.

Picture sharpness alone rarely was an issue whereby we infer that to improve the success rate of filming, more emphasis should be placed on parental training in future studies. In our study, only nine participants received hands-on assistance in installation of the app at the hospital, and the rest were guided remotely by phone. A follow-up by phone at six weeks' age was added to reduce attrition, and telephone contact was made in most cases at the time of filming. Kwong et al. in Australia recently demonstrated that simple instructions for filming could potentially replace a separate smartphone app for achieving good results with remote GMA.[27] This approach has also shown promise in a small pilot study in India.[28] However, the potential benefit of separate apps

For peer review only - http://bmjopen.bmj.com/site/about/guidelines.xhtml

such as NeuroMotion™ is the possibility of ensuring safe data transfer, as privacy regulations need to be considered when implementing telemedicine interventions.[14]

Parents participating in the study had positive experiences with the NeuroMotion™ app and the follow-up in general. The response rate to the usability survey was higher than that in previous studies, and we also interviewed parents who had failed to film their child as intended. The results were similar to those in HIC settings, with Nepalese and Australian parents showing higher rates of worry and preference for physical examination than the other two studies.[16,17,24] A separate qualitative study will explore the experiences of the participating parents in-depth (manuscript submitted), but future implementation should consider ways to alleviate parental stress during the follow-up.

Realising the aim of the Sustainable Development Goals agenda of leaving no child behind will only be achieved if remote GMA screening is conducted as part of a follow-up package with simultaneous improvement in pre- and peripartum care. The validity of GMA in LMIC settings is yet to be proven, as most studies conducted thus far have been small like ours.[29] Broad developmental assessment should be provided to children at risk of disability as the GMA has poorer predictive ability for other adverse neurodevelopmental outcomes than CP.[30] A report from Australia published during the Covid-19 pandemic showed that GMA analysis could be performed during a Zoom call as a part of a comprehensive developmental evaluation.[31] In LMIC settings, GMA could be added to risk child follow-up by engaging community health workers in filming, which might improve the quality of the films. In India, an on-going trial is evaluating the possibility of teaching community health workers the GMA method,[3] and in the future, automatized analysis could help in mass screening of videos when filming is done in a standardized manner.[18]

Finally, ensuring linkage to an early intervention program is imperative for ethical conduct of GMA screening. In our study, three children at high risk of CP with absent FMs were identified, but one of the families moved abroad, and the other two were not willing to enrol in the offered free intervention program. The ability of GMA to identify children at high risk of CP before overt signs of the condition appear can be a drawback in settings such as Nepal, where there is no tradition for postnatal follow-up and parents have limited resources available. Furthermore, care seeking from alternative providers is common in Nepal as CP is perceived to be caused by also other than medical reasons.[32,33] Further qualitative studies might shed light on the appropriate ways of informing parents about the diagnosis of high-risk CP in low- and middle-income settings.[34] Early intervention conducted partially via a digital platform might also increase uptake considering the overall positive attitudes parents showed towards remote screening.

Limitations

This study was a small single-centre feasibility study conducted in the capital city of Nepal with a relatively high standard of living, education and mobile phone access, and the results are thus not generalizable to rural areas of the country. Despite extending the recruitment period, we did not reach the planned sample size. The study was not powered to properly examine background predictors that might have impacted filming success, and it is essential that alternative means of

For peer review only

assessment are provided parallel to remote assessment to avoid discriminating disadvantaged groups such as females, who only represented 20% of the enrolled infants.

We attempted to minimize participation bias by interviewing parents who did not succeed in filming. However, as the interview was conducted by phone by the same research assistant who had provided guidance regarding the app installation and the results of the GMA analysis, it is possible that social desirability bias influenced parental responses.

Finally, the study did not assess the acceptability of remote GMA from the provider point of view, a necessary consideration along with legislation and infrastructure for future scale-up of the intervention.[14]

Conclusion

Engaging parents in screening of CP with the help of remote GMA is possible in the urban area of a mountainous South Asian lower middle-income country and is generally welcomed by the participants, but scale-up will require substantial resources.

References

1. World Health Organization. Nurturing care for early childhood development: a framework for helping children survive and thrive to transform health and human potential. World Health Organization, United Nations Children's Fund, World Bank Group; 2018.

2. Morgan C, Fetters L, Adde L, Badawi N, Bancale A, Boyd RN, et al. Early Intervention for Children Aged 0 to 2 Years With or at High Risk of Cerebral Palsy: International Clinical Practice Guideline Based on Systematic Reviews. JAMA Pediatr. 2021 Aug 1;175(8):846–58.

3. Toldo M, Varishthananda S, Einspieler C, Tripathi N, Singh A, Verma SK, et al. Enhancing early detection of neurological and developmental disorders and provision of intervention in low-resource settings in Uttar Pradesh, India: study protocol of the G.A.N.E.S.H. programme. BMJ Open. 2020 Nov 1;10(11):e037335.

4. Benfer KA, Novak I, Morgan C, Whittingham K, Khan NZ, Ware RS, et al. Community-based parent-delivered early detection and intervention programme for infants at high risk of cerebral palsy in a low-resource country (Learning through Everyday Activities with Parents (LEAP-CP): protocol for a randomised controlled trial. BMJ Open. 2018 Jun;8(6):e021186.

5. Nanyunja C, Sadoo S, Kohli-Lynch M, Nalugya R, Nyonyintono J, Muhumuza A, et al. Early care and support for young children with developmental disabilities and their caregivers in Uganda: The Baby Ubuntu feasibility trial. Front Pediatr. 2022 Sep 13;10:981976.

For peer review only - http://bmjopen.bmj.com/site/about/guidelines.xhtml

6. McIntyre S, Goldsmith S, Webb A, Ehlinger V, Hollung SJ, McConnell K, et al. Global prevalence of cerebral palsy: A systematic analysis. Developmental Medicine & Child Neurology. 2022;64(12):1494–506.

7. Scherzer AL, Chhagan M, Kauchali S, Susser E. Global perspective on early diagnosis and intervention for children with developmental delays and disabilities. Dev Med Child Neurol. 2012 Dec;54(12):1079–84.

8. Al Imam MH, Jahan I, Muhit M, Hardianto D, Laryea F, Chhetri AB, et al. Predictors of Rehabilitation Service Utilisation among Children with Cerebral Palsy (CP) in Low- and Middle-Income Countries (LMIC): Findings from the Global LMIC CP Register. Brain Sciences. 2021 Jun 25;11(7):848.

9. Einspieler C, Prechtl HFR, Bos AF, Ferrari F, Cioni G. Prechtl's Method on the Qualitative Assessment of General Movements in Preterm, Term and Young Infants. Cambridge University Press; 2005. 104 p.

10. Kwong AKL, Fitzgerald TL, Doyle LW, Cheong JLY, Spittle AJ. Predictive validity of spontaneous early infant movement for later cerebral palsy: a systematic review. Dev Med Child Neurol. 2018;60(5):480–9.

11. Novak I, Morgan C, Adde L, Blackman J, Boyd RN, Brunstrom-Hernandez J, et al. Early, Accurate Diagnosis and Early Intervention in Cerebral Palsy: Advances in Diagnosis and Treatment. JAMA Pediatr. 2017 Sep 1;171(9):897–907.

12. Tomantschger I, Herrero D, Einspieler C, Hamamura C, Voos MC, Marschik PB. The general movement assessment in non-European low- and middle-income countries. Rev Saude Publica. 2018 Jan 29;52(6):1–8.

13. Fleming KA, Horton S, Wilson ML, Atun R, DeStigter K, Flanigan J, et al. The Lancet Commission on diagnostics: transforming access to diagnostics. The Lancet. 2021;398(10315):1997–2050.

14. World Health Organization. WHO guideline recommendations on digital interventions for health system strengthening. 2019;

15. Spittle A, Olsen J, Kwong A, Doyle L, Marschik P, Einspieler C, et al. The Baby Moves prospective cohort study protocol: using a smartphone application with the General Movements Assessment to predict neurodevelopmental outcomes at age 2 years for extremely preterm or extremely low birthweight infants. BMJ open. 2016;6(10):e013446.

16. Adde L, Brown A, Van Den Broeck C, Decoen K, Eriksen BH, Fjørtoft T, et al. In-Motion-App for remote General Movement Assessment: A multi-site observational study. BMJ Open. 2021;11(3).

For peer review only

17. Svensson KA, Örtqvist M, Bos AF, Eliasson AC, Sundelin HEK. Usability and inter-rater reliability of the NeuroMotion app: A tool in General Movements Assessments. European Journal of Paediatric Neurology. 2021 Jul;33:29–35.

18. Silva N, Zhang D, Kulvicius T, Gail A, Barreiros C, Lindstaedt S, et al. The future of General Movement Assessment: The role of computer vision and machine learning - A scoping review. Res Dev Disabil. 2021 Mar;110:103854.

19. Vandenbroucke JP, Elm E von, Altman DG, Gøtzsche PC, Mulrow CD, Pocock SJ, et al. Strengthening the Reporting of Observational Studies in Epidemiology (STROBE): Explanation and Elaboration. PLOS Medicine. 2007 Oct 16;4(10):e297.

20. Eldridge SM, Chan CL, Campbell MJ, Bond CM, Hopewell S, Thabane L, et al. CONSORT 2010 statement: extension to randomised pilot and feasibility trials. Pilot Feasibility Stud. 2016;2:64.

21. Lancaster GA, Thabane L. Guidelines for reporting non-randomised pilot and feasibility studies. Pilot and Feasibility Studies. 2019 Oct 6;5(1):114.

22. Ministry of Health and Population, Nepal, New ERA, Kathmandu, ICF. Nepal Demographic and Health Survey 2022: Key Indicators Report. Ministry of Health and Population, Kathmandu, Nepal; 2022.

23. Kc A, Wrammert J, Clark RB, Ewald U, Vitrakoti R, Chaudhary P, et al. Reducing Perinatal Mortality in Nepal Using Helping Babies Breathe. Pediatrics. 2016;137(6).

24. Kwong AK, Eeles AL, Olsen JE, Cheong JL, Doyle LW, Spittle AJ. The Baby Moves smartphone app for General Movements Assessment: Engagement amongst extremely preterm and term-born infants in a state-wide geographical study. J Paediatr Child Health. 2018 Oct 4;

25. Proctor E, Silmere H, Raghavan R, Hovmand P, Aarons G, Bunger A, et al. Outcomes for implementation research: Conceptual distinctions, measurement challenges, and research agenda. Administration and Policy in Mental Health and Mental Health Services Research. 2011;38(2):65–76.

26. Hayes A, Krippendorff K. Answering the Call for a Standard Reliability Measure for Coding Data. Communication Methods and Measures. 2007 Apr 1;1:77–89.

27. Kwong AKL, Eeles AL, Olsen JE, Zannino D, Kariotis T, Spittle AJ. Instructional guides for filming infant movements at home are effective for the General Movements Assessment. Journal of Paediatrics and Child Health. 2021;58(5):796–801.

28. Saini L, Madaan P, Bhagwat C, Einspieler C. Home-videos for neurodevelopmental follow-up of high-risk infants during COVID-19 pandemic: a simple and inexpensive tool. Journal of Tropical Pediatrics. 2021;67(1):fmaa088.

For peer review only

For peer review only - http://bmjopen.bmj.com/site/about/guidelines.xhtml

29. King AR, Al Imam MH, McIntyre S, Morgan C, Khandaker G, Badawi N, et al. Early Diagnosis of Cerebral Palsy in Low- and Middle-Income Countries. Brain Sci. 2022 Apr 23;12(5):539.

30. Caesar R, Colditz PB, Cioni G, Boyd RN. Clinical tools used in young infants born very preterm to predict motor and cognitive delay (not cerebral palsy): a systematic review. Dev Med Child Neurol. 2021 Apr;63(4):387–95.

31. Maitre NL, Benninger KL, Neel ML, Haase JA, Pietruszewski L, Levengood K, et al. Standardized Neurodevelopmental Surveillance of High-risk Infants Using Telehealth: Implementation Study during COVID-19. Pediatr Qual Saf. 2021 Jul 28;6(4):e439.

32. Saul R, Phillips D. Ghosts and germs: Cerebral Palsy in Nepal: A preliminary exploration of cosmology and disability. Disability and development: Learning from action and research on disability in the majority world. 1999;

33. Thapa R. Symptom Recognition and Diagnosis of Cerebral Palsy in Nepal. J Autism Dev Disord. 2017;47(6):1739–48.

34. Byrne R, Duncan A, Pickar T, Burkhardt S, Boyd RN, Neel ML, et al. Comparing parent and provider priorities in discussions of early detection and intervention for infants with and at risk of cerebral palsy. Child: Care, Health and Development. 2019;45(6):799–807.

Author statement:

AJK, AKC, HEKS, HL, JW, KUS, NJWB, PP and RG designed the study.
AJK, AKC, KUS, OB, PB, PP and RG collected the data and/or supervised data acquisition.
AJK, HEKS, KS and OB analysed the data.
AJK drafted the manuscript and had access to all the data. All authors participated in revising the manuscript critically.
All authors approve the submitted version.

Acknowledgements: Asmita Basnet recruited the study participants and guided the parents in installing the NeuroMotion™ app in the hospital. Rabina Karki conducted the phone follow-up, provided support for the parents during filming and administered the usability survey. Anna Axelin contributed to the design of the study. The authors wish to thank Alicia Spittle and Amanda Kwon for permission to use the usability survey questionnaire.

For peer review only - http://bmjopen.bmj.com/site/about/guidelines.xhtml

Figure and Table Legends

Figure 1: Participant flow diagram. FM =Fidgety Movements, SNCU/NICU = Sick Newborn Care Unit/Neonatal Intensive Care Unit

Figure 2: Stacked bar graph of parental answers to smartphone app usability survey. Comparison of answers between the BabyMoves app in Australia,[24] In-Motion app in Belgium, Denmark and Norway,[16] NeuroMotion app in Sweden[17] and NeuroMotion app Nepal (current study).

Table 1: Background characteristics. Comparison between those participants who returned at least one film of acceptable technical quality and those who did not.

For peer review only

For peer review only - http://bmjopen.bmj.com/site/about/guidelines.xhtml

Supporting information

Supplementary Table 1: Results of the video quality assessment and evaluation of Fidgety Movements.

Supplementary Material: GMA (GM trust) specialist assessment of NeuroMotion films

For peer review only

For peer review only - http://bmjopen.bmj.com/site/about/guidelines.xhtml

Table 1:

	Filmed successfully = 16	Not filmed successfully = 15	p-value	cOR (95%CI)
INFANT CHARACTERISTICS				
Gestational age in weeks, Mean (SD)	38.9 (1.4)	38.8 (1.5)	0.32*	-
Apgar at 5 min, Mean (SD)	5.3 (0.4)	4.8 (0.7)	0.67*	-
Resuscitation at 5 min	16	14	0.29**	NA
Clinical seizures	3	7	0.14***	0.26 (0.05-1.32)
Sex			1.00***	
Female	3	3		Ref
Male	13	12		1.08 (0.18-6.42)
Mode of delivery			0.25***	
Normal vaginal	13	9		Ref
Instrumental	0	0		-
Caesarean section	3	6		0.34 (0.06-1.75)
DEMOGRAPHIC FACTORS				
Maternal education			0.36***	
1-7 years	5	3		Ref

For peer review only - http://bmjopen.bmj.com/site/about/guidelines.xhtml

	Filmed successfully = 16	Not filmed successfully = 15	p-value	cOR (95%CI)
None	0	2		-
Complete primary	3	2		0.45 (0.05-3.57)
Complete secondary	3	6		0.30 (0.04-2.20-
University degree	5	2		1.50 (0.17-13.22)
**Maternal ethnicity**			0.90**	
Relatively disadvantageous	11	10		Ref
Relatively advantageous	5	5		1.10 (0.24-4.96)
**Maternal age, Mean (SD)**	25y5m (3y6m)	24y2m (3y)	0.60*	
**Paternal ethnicity**			0.90**	
Relatively disadvantageous	11	10		Ref
Relatively advantageous	5	5		1.10 (0.24-4.96)
**Paternal age, Mean (SD)**	29y6m (4y7m)	27y7m (3y3m)	0.37*	

For peer review only

	Filmed successfully = 16	Not filmed successfully = 15	p-value	cOR (95%CI)
SMARTPHONE USE				
Parents own a smart phone	16	15	NA	-
Years with smart phone, Mean (SD)	9y1m (3y2m)	7y7m (3y)	0.68*	-
Parents use social media	15	15	0.33**	NA
App installation			1.00***	
Hospital	5	4	1.00	Ref
Home	11	11	1.00	0.80 (0.16-3.79)

CI = Confidence Interval, cOR = crude Odds Ratio, SD = Standard Deviation. No missing data.

*independent samples t-test, ** Pearson's chi-square test if a cell has count 5 or more, ***Fischer's exact text if a cell has count less than 5. Parental ethnicity was self-reported. Relatively disadvantageous = Dalit, Janajati, Madhesi or Muslim, Relatively advantageous = Brahmini, Chhetri or other.

For peer review only

For peer review only - http://bmjopen.bmj.com/site/about/guidelines.xhtml

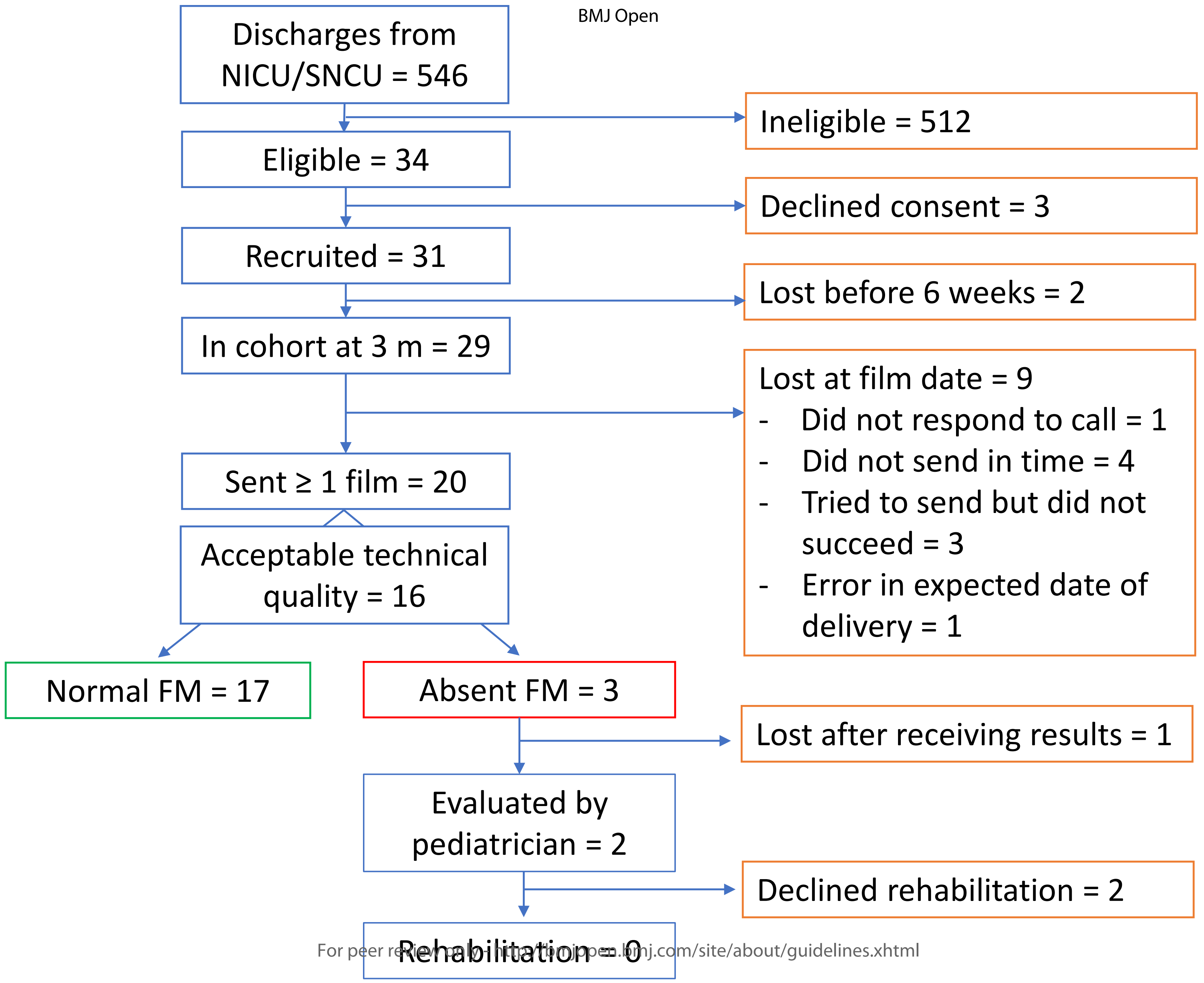
Discharges from NICU/SNCU = 546
Ineligible = 512
Eligible = 34
Declined consent = 3
Recruited = 31
Lost before 6 weeks = 2
In cohort at 3 m = 29
Lost at film date = 9
- Did not respond to call = 1
- Did not send in time = 4
- Tried to send but did not succeed = 3
- Error in expected date of delivery = 1
Sent ≥ 1 film = 20
Acceptable technical quality = 16
Normal FM = 17
Absent FM = 3
Lost after receiving results = 1
Evaluated by pediatrician = 2
Declined rehabilitation = 2
Rehabilitation = 0

**Person answering the survey**	
Mother	23
Father	2
**Respondent present during filming**	
Yes	23
No	2
**Type of smartphone used**	
Android	23
iPhone	1
Not known	1

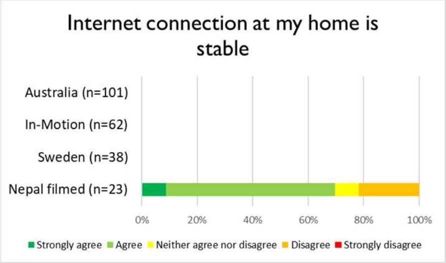

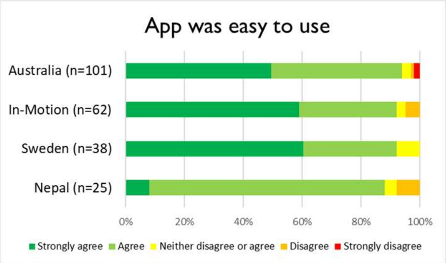

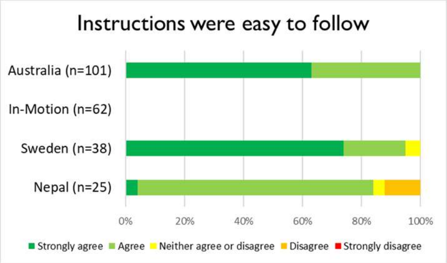

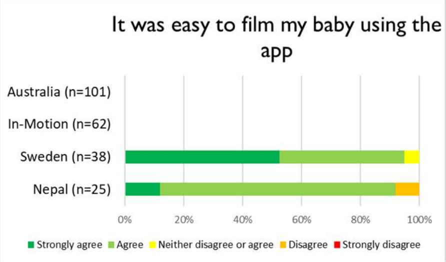

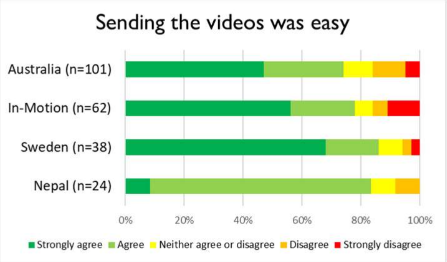

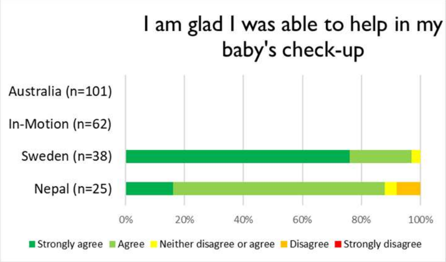

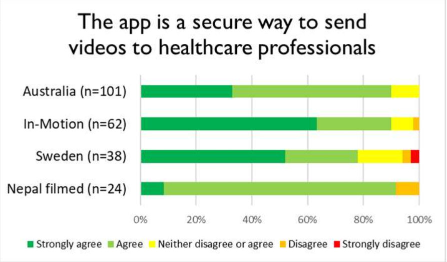

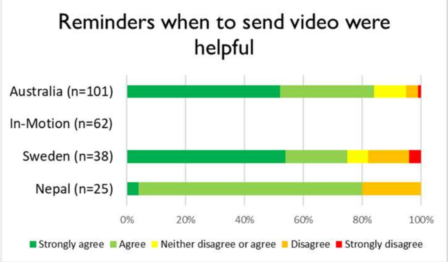

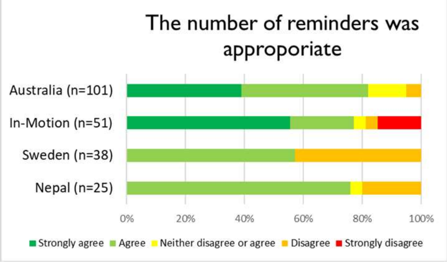

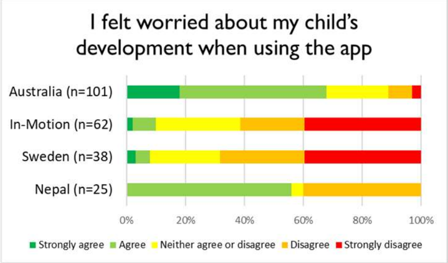

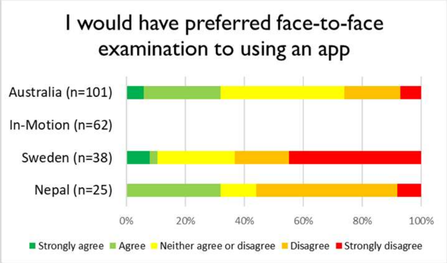

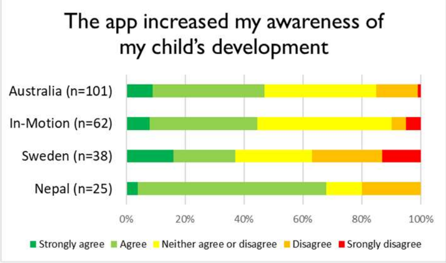

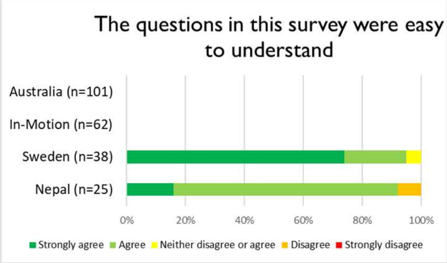

		Picture quality			Baby setting					Overall judgement	Fidgety Movements
Film number	Case number	Sharpness	Camera held in correct pos.	Camera held steady	Clothing	Background/ blanket	Supine position	Surrounding influences	Baby's mood		
1	1	G	E	G	E	E	E	E	E	Approved	F-
2	1	G	I	G	E	E	E	E	E	Approved	F-
3	1	G	G	G	E	E	E	E	E	Approved	F-
4	2	E	I	I	E	G	E	E	E	Approved	F+
5	2	E	I	I	E	E	E	E	E	Rejected	?
6	2	E	I	I	E	G	E	E	E	Approved	F+
7	3	E	E	I	E	G	E	E	E	Approved	F+
8	4	E	E	G	E	G	E	E	E	Approved	F+
9	4	E	E	G	E	G	E	E	E	Approved	F+
10	4	E	E	G	E	G	E	G	E	Approved	F+
11	4	E	E	G	E	G	G	I	E	Rejected	F+
12	4	E	I	I	E	G	G	I	E	Rejected	?
13	4	E	E	E	E	G	E	G	E	Approved	F+
14	5	E	E	E	G	E	E	E	E	Approved	F+
15	5	E	E	E	G	I	E	E	E	Approved	F+
16	5	E	G	E	E	G	E	E	E	Approved	F+
17	5	E	E	E	E	E	E	E	E	Approved	F+
18	5	E	E	E	E	E	E	E	E	Approved	F+
19	6	E	E	I	E	E	E	E	E	Approved	F+
20	6	E	E	G	E	E	E	E	E	Approved	F+
21	7	E	E	G	E	I	E	I	G	Approved	F+
22	7	E	E	I	G	I	E	E	E	Approved	F+
23	8	E	I	I	G	G	E	I	E	Rejected	F+
24	9	G	G	G	E	I	E	G	E	Approved	F+
25	10	E	G	G	E	G	E	G	E	Approved	F-
26	10	G	E	I	E	I	E	I	E	Approved	F-
27	10	E	G	I	E	G	E	G	E	Approved	F-
28	11	E	I	I	I	I	E	E	E	Rejected	?
29	11	E	I	I	E	E	E	I	E	Rejected	F+
30	12	I	E	G	I	G	E	E	E	Rejected	?
31	12	I	G	G	I	I	G	E	E	Rejected	F+
32	12	E	G	E	G	E	E	E	E	Approved	F+
33	12	E	G	G	I	I	E	I	E	Approved	F+
34	13	E	G	G	E	E	E	E	E	Approved	F+
35	13	E	E	G	E	E	E	E	E	Approved	F+
36	14	E	I	I	E	I	E	I	E	Rejected	F-(?)
37	15	E	G	G	E	E	E	E	E	Approved	F+

For peer review only

38	16	E	I	I	I	I	E	I	E	Rejected	?
39	16	E	E	E	G	E	E	E	E	Approved	F+
40	16	E	E	E	G	E	E	E	E	Approved	F+
41	17	E	E	E	E	E	E	E	E	Approved	F+
42	18	E	E	E	E	E	E	E	E	Approved	F+
43	18	E	E	E	E	E	E	G	E	Approved	F+
44	19	E	G	E	E	E	E	E	E	Approved	F+
45	19	E	I	G	E	G	E	E	E	Approved	F+
46	20	E	I	I	E	I	E	E	E	Rejected	F+
Summary of results	Excellent	39	23	13	34	20	43	31	45		
	Good	5	11	18	7	15	3	6	1		
	Indistinct	2	12	15	5	11	0	9	0		
Films number 11, 23, 29 and 31 were rejected for technical quality, but Fidgety Movements were clearly observed.											
Case number 14 only sent one film that was rejected due to poor technical quality, but Fidgety Movements were not observed.											

Supplementary Table 1: Results of the video quality assessment and evaluation of Fidgety Movements.

For peer review only

Supplementary Material: GMA (GM trust) specialist assessment of NeuroMotion films

Film #

Assessed by:________________________________

Date:________________________________

Quality	Approved		Not approved	Comments
	Excellent	Good	Indistinct	
Picture quality				
Sharpness				
Camera is held in correct position				
Camera is held in a steady position				
Baby setting				
Clothing				
Background/blanket				
Supine position				
Surrounding influences				
Baby´s mood				
Overall judgement:				

For peer review only

STROBE Statement—Checklist of items that should be included in reports of ***cohort studies***

	Item No	Recommendation	
**Title and abstract**	1	(*a*) Indicate the study's design with a commonly used term in the title or the abstract	1
		(*b*) Provide in the abstract an informative and balanced summary of what was done and what was found	2
**Introduction**			
Background/rationale	2	Explain the scientific background and rationale for the investigation being reported	3
Objectives	3	State specific objectives, including any prespecified hypotheses	5
**Methods**			
Study design	4	Present key elements of study design early in the paper	3
Setting	5	Describe the setting, locations, and relevant dates, including periods of recruitment, exposure, follow-up, and data collection	3-5
Participants	6	(*a*) Give the eligibility criteria, and the sources and methods of selection of participants. Describe methods of follow-up	4
		(*b*) For matched studies, give matching criteria and number of exposed and unexposed	N/A
Variables	7	Clearly define all outcomes, exposures, predictors, potential confounders, and effect modifiers. Give diagnostic criteria, if applicable	5
Data sources/ measurement	8*	For each variable of interest, give sources of data and details of methods of assessment (measurement). Describe comparability of assessment methods if there is more than one group	4,5
Bias	9	Describe any efforts to address potential sources of bias	8
Study size	10	Explain how the study size was arrived at	4
Quantitative variables	11	Explain how quantitative variables were handled in the analyses. If applicable, describe which groupings were chosen and why	5, table 1
Statistical methods	12	(*a*) Describe all statistical methods, including those used to control for confounding	5
		(*b*) Describe any methods used to examine subgroups and interactions	N/A
		(*c*) Explain how missing data were addressed	N/A
		(*d*) If applicable, explain how loss to follow-up was addressed	not relevant
		(*e*) Describe any sensitivity analyses	N/A
**Results**			
Participants	13*	(a) Report numbers of individuals at each stage of study—eg numbers potentially eligible, examined for eligibility, confirmed eligible, included in the study, completing follow-up, and analysed	Fig 1
		(b) Give reasons for non-participation at each stage	Fig 1
		(c) Consider use of a flow diagram	Fig 1
Descriptive data	14*	(a) Give characteristics of study participants (eg demographic, clinical, social) and information on exposures and potential confounders	Table 1
		(b) Indicate number of participants with missing data for each variable of interest	table 1
		(c) Summarise follow-up time (eg, average and total amount) N/A	ingress
Outcome data	15*	Report numbers of outcome events or summary measures over time	
Main results	16	(*a*) Give unadjusted estimates and, if applicable, confounder-adjusted estimates and their precision (eg, 95% confidence interval). Make clear which confounders were adjusted for and why they were included only cOR reported	
		(*b*) Report category boundaries when continuous variables were categorized	not relevant
		(*c*) If relevant, consider translating estimates of relative risk into absolute risk for a meaningful time period	

For peer review only

Other analyses	17	Report other analyses done—eg analyses of subgroups and interactions, and sensitivity analyses	N/A
**Discussion**			
Key results	18	Summarise key results with reference to study objectives	7
Limitations	19	Discuss limitations of the study, taking into account sources of potential bias or imprecision. Discuss both direction and magnitude of any potential bias	8
Interpretation	20	Give a cautious overall interpretation of results considering objectives, limitations, multiplicity of analyses, results from similar studies, and other relevant evidence	7
Generalisability	21	Discuss the generalisability (external validity) of the study results	8
**Other information**			
Funding	22	Give the source of funding and the role of the funders for the present study and, if applicable, for the original study on which the present article is based	12

*Give information separately for exposed and unexposed groups.

Note: An Explanation and Elaboration article discusses each checklist item and gives methodological background and published examples of transparent reporting. The STROBE checklist is best used in conjunction with this article (freely available on the Web sites of PLoS Medicine at http://www.plosmedicine.org/, Annals of Internal Medicine at http://www.annals.org/, and Epidemiology at http://www.epidem.com/). Information on the STROBE Initiative is available at http://www.strobe-statement.org.

For peer review only

For peer review only - http://bmjopen.bmj.com/site/about/guidelines.xhtml

BMJ Open

NeuroMotion™ Smartphone Application for Remote General Movements Assessment: a Feasibility Study in Nepal

Journal:	*BMJ Open*
Manuscript ID	bmjopen-2023-080063.R1
Article Type:	Original research
Date Submitted by the Author:	19-Dec-2023
Complete List of Authors:	Kukka, Antti; Uppsala University, Department of Women’s and Children’s Health; Region Gävleborg, Department of Pediatrics<br>Sundelin, Heléne; Linköping University, Department of Biomedical and Clinical Sciences; Karolinska Institutet, Department of Women's and Children's Health<br>Basnet, Omkar; Golden Community<br>Paudel, Prajwal; Paropakar Maternity and Women’s Hospital<br>Subedi, Kalpana; Paropakar Maternity and Women's Hospital<br>Svensson, Katarina; Linköping University, Division of Children's and Women's Health, Department of Biomedical and Clinical Sciences<br>brown, nick; Uppsala Universitet, Department of Women's and Children's Health; Region Gävleborg, Department of Pediatrics<br>Litorp, Helena; Uppsala University, Department of Women’s and Children’s Health; Karolinska Institutet, Department of Global Public Health<br>Gurung, Rejina; Uppsala University, Department of Women’s and Children’s Health; Golden Community<br>Bhattarai, Pratiksha; Golden Community<br>Wrammert, Johan; Uppsala University, Department of Women’s and Children’s Health<br>Ashish, KC; Uppsala University, Department of Women’s and Children’s Health; University of Gothenburg, School of Public Health and Community Medicine
**Primary Subject Heading**:	Paediatrics
Secondary Subject Heading:	Neurology, Health informatics, Global health, Communication, Diagnostics
Keywords:	Developmental neurology & neurodisability < PAEDIATRICS, Feasibility Studies, Nepal, Follow-Up Studies, NEONATOLOGY, eHealth

SCHOLARONE™
Manuscripts

BMJ

I, the Submitting Author has the right to grant and does grant on behalf of all authors of the Work (as defined in the below author licence), an exclusive licence and/or a non-exclusive licence for contributions from authors who are: i) UK Crown employees; ii) where BMJ has agreed a CC-BY licence shall apply, and/or iii) in accordance with the terms applicable for US Federal Government officers or employees acting as part of their official duties; on a worldwide, perpetual, irrevocable, royalty-free basis to BMJ Publishing Group Ltd ("BMJ") its licensees and where the relevant Journal is co-owned by BMJ to the co-owners of the Journal, to publish the Work in this journal and any other BMJ products and to exploit all rights, as set out in our licence.

The Submitting Author accepts and understands that any supply made under these terms is made by BMJ to the Submitting Author unless you are acting as an employee on behalf of your employer or a postgraduate student of an affiliated institution which is paying any applicable article publishing charge ("APC") for Open Access articles. Where the Submitting Author wishes to make the Work available on an Open Access basis (and intends to pay the relevant APC), the terms of reuse of such Open Access shall be governed by a Creative Commons licence – details of these licences and which Creative Commons licence will apply to this Work are set out in our licence referred to above.

Other than as permitted in any relevant BMJ Author's Self Archiving Policies, I confirm this Work has not been accepted for publication elsewhere, is not being considered for publication elsewhere and does not duplicate material already published. I confirm all authors consent to publication of this Work and authorise the granting of this licence.

For peer review only

NeuroMotion™ Smartphone Application for Remote General Movements Assessment: a Feasibility Study in Nepal

Author list:

Antti J. Kukka[1,2], Heléne E.K. Sundelin[3,4], Omkar Basnet[5], Prajwal Paudel[6], Kalpana Upadhyaya Subedi[6], Katarina Svensson[3,7], Nick J.W. Brown[1,2], Helena Litorp[1,8], Rejina Gurung[1,5], Pratiksha Bhattarai[5], Johan Wrammert[1] and Ashish KC[1,9]

Affiliations:

1. Department of Women's and Children's Health, Uppsala University, Sweden
2. Department of Pediatrics, Gävle Regional Hospital, Region Gävleborg, Sweden
3. Division of Children´s and Women´s Health, Department of Biomedical and Clinical Sciences, Linköping University, Linköping, Sweden.
4. Neuropediatric Unit, Department of Women's and Children's Health, Karolinska University Hospital, Karolinska Institutet, Stockholm, Sweden
5. Golden Community, Lalitpur, Nepal
6. Paropakar Maternity and Women's Hospital, Kathmandu, Nepal
7. Crown Princess Victoria's Children's and Youth Hospital, University Hospital, Linkoping, Sweden
8. Department of Global Public Health, Karolinska Institutet, Stockholm, Sweden
9. School of Public Health and Community Medicine, University of Gothenburg, Gothenburg, Sweden

Corresponding author: Antti Kukka, Department of Women's and Children's Health, Uppsala University, SE-751 85, Uppsala, Sweden, https://www.katalog.uu.se/profile/?id=N19-2319
Email address: antti.kukka@uu.se

ORCID:

Antti Juhani Kukka https://orcid.org/0000-0002-5879-7417
Heléne Sundelin https://orcid.org/0000-0002-8189-4098
Omkar Basnet https://orcid.org/0000-0001-7328-8189
Prajwal Paudel
Kalpana Subedi https://orcid.org/0000-0002-2019-1997
Katarina Svensson https://orcid.org/0000-0001-9368-8366
Nick Brown https://orcid.org/0000-0003-1789-0436
Helena Litorp https://orcid.org/0000-0001-7164-598X
Rejina Gurung 0000-0002-4262-3543
Pratiksha Bhattarai https://orcid.org/0000-0001-9455-4394
Johan Wrammert orcid.org/0000-0002-9430-7681
Ashish KC https://orcid.org/0000-0002-0541-4486

For peer review only

Abbreviations:

CP = cerebral palsy
FM = fidgety movements
GMA = General Movements Assessment
HIC = high-income country
NICU/SNCU = neonatal intensive care unit/sick newborn care unit

Abstract:

OBJECTIVES: To evaluate the feasibility of using the NeuroMotion™ smartphone application for remote General Movements Assessment for screening of infants for cerebral palsy in Kathmandu, Nepal.

METHOD: Thirty-one term born infants at risk of cerebral palsy due to birth asphyxia or neonatal seizures were recruited for the follow-up at Paropakar Maternity and Women's Hospital, 1st October 2021 to 7th January 2022. Parents filmed their children at home using the app at 3 months' age and the videos were assessed for technical quality using a standardized form and for fidgety movements by Prechtl's General Movements Assessment. Usability of the app was evaluated through a parental survey.

RESULTS: Twenty families sent in altogether 46 videos out of which 35 had approved technical quality. Sixteen children had at least one video with approved technical quality. Three infants lacked fidgety movements. The level of agreement between assessors was acceptable (Krippendorf alpha 0.781). Parental answers to the usability survey were in general positive.

INTERPRETATION: Engaging parents in screening of cerebral palsy with the help of a smartphone-aided remote General Movements Assessment is possible in the urban area of a South Asian lower middle-income country.

Short title: Screening of CP using remote GMA in Nepal

Word count: 3093

Keywords: cerebral palsy, general movements assessment, neonatal follow-up, low- and middle-income countries, pilot projectsArticle summary

Strengths and limitations of this study:

- Three independent assessors judged the presence of General Movements in each video.
- Standardized assessment of the technical quality of videos was performed.
- Social desirability bias might have contributed to the positive results in the usability survey.
- Small sample from a single referral hospital limits generalizability of the findings.

For peer review only

For peer review only - http://bmjopen.bmj.com/site/about/guidelines.xhtml

Background

All children with disability have the right reach their full developmental potential [1]. Cerebral palsy (CP) is the most common motor disability of childhood and international guidelines recommend early intervention for all children at high risk of CP [2]. A number of on-going trials are evaluating ways to adapt early intervention to low- and middle-income countries (LMICs) [3-5], which shoulder the vast majority of children affected by CP globally [6]. Delay in accessing rehabilitation is common due to poor availability and lack of targeted screening programs for high risk children [7-8].

Affordable and accurate tools are therefore needed to identify children at high risk of CP in LMICs. General Movements Assessment (GMA) is a cheap, non-invasive method for recognizing these children based on observing their spontaneous movements [9]. It has been shown to have excellent validity at 3 months' age and its use in screening of high risk children is now globally endorsed [10-11], although its accuracy in LMIC populations is yet to be established [12]. Lack of providers trained in the methodology has thus far limited its implementation [13], and telemedicine is one way of overcoming this barrier [14,15]. Lockdowns imposed during the recent Covid-19 pandemic added urgency for finding alternatives for traditional face-to-face check-ups.

Previous studies in high-income countries (HICs) have shown that remote GMA using smartphone applications (apps) is feasible [16-18]. Automatized movement analysis using machine learning holds further promise for wider availability [19]. However, to our knowledge no previous study has utilized smartphone apps for remote GMA in LMIC settings, where the disease burden is the greatest. We therefore aimed to evaluate the feasibility of using smartphone aided remote GMA for screening of infants at risk of CP in Kathmandu, the capital of Nepal, a lower middle-income country. Feasibility was assessed through following specific objectives:

1) to estimate the *reach* of the screening program

2) to evaluate the *technical quality* of the videos and *quality of the assessment*

3) to survey parental perceptions of the *usability* of the app.

Methods

A combination of the STROBE checklist for cohort studies [20] and CONSORT statement for pilot and feasibility trials [21] was used for reporting the study following Guidelines for reporting non-randomised pilot and feasibility studies [22].

Study design and setting

This is a single-centre feasibility study conducted at the Paropakar Maternity and Women's Hospital, Kathmandu, Nepal. Recruitment of infants ran from 1st October 2021 to 7th January 2022 just before the onset of third peak of the Covid-19 pandemic and last response to the usability

For peer review only - http://bmjopen.bmj.com/site/about/guidelines.xhtml

survey was registered on 2nd March 2023. Paropakar is a public tertiary hospital acting as the referral hospital for the surrounding Bagmati province. Infants admitted to the hospital's Neonatal Intensive Care Unit/Sick Newborn Care Unit (NICU/SNCU) are offered post-discharge follow-up up at the paediatric outpatient department up to 14 years' age, but few families choose to participate.

Nationally only 70 % of infants receive a post-natal check-up in Nepal [23].Targeted developmental clinics for high risk children are generally lacking whereby delay in diagnosis until nearly school age is common [24,25]. Paediatric rehabilitation services are for CP are provided by non-governmental organizations and government hospitals working by Community Based Rehabilitation principles [26]. Ownership of smartphones in the Bagmati province is 75 and 84 % among women and men respectively [23].

Participants

Term born neonates (≥ 37 completed gestational weeks according to last menstrual period) at risk of CP due to birth asphyxia or seizures were recruited to the follow-up. The included infants had to survive to discharge and fulfil at least one of the following criteria: 1) Apgar score ≤ 5 at 5 minutes; 2) bag-and-mask ventilation ≥ 5 minutes after delivery; or 3) diagnosis of clinical seizures at the NICU/SNCU.

Newborn infants who were transferred to other hospitals, and those whose parents lived outside of the Kathmandu valley with travel time to the hospital above one hour were excluded due to limited availability of rehabilitation resources outside the capital.

All infants potentially eligible for the study were routinely admitted to the NICU/SNCU, which was confirmed by a chart review of 3 preceding months. Paediatricians working at the wards identified eligible infants and informed a research assistant in charge of recruitment. Background demographic data and delivery details were collected by a short parental interview and patient chart review respectively. All participating parents were contacted by a phone call when their child was six weeks old to enquire about the health of the child and to give parents a possibility to ask further questions about the study.

Sample size

In preparation for this study, we calculated that to show the sensitivity and specificity of GMA for CP diagnosis at two years of age with 95% certainty when the expected sensitivity and specificity are 95% and the expected proportion of infants with abnormal outcome in the population is 20%, then using Buderer's formula [27], 365 infants would need to be recruited. A final sample size of 400 would have been needed to account for losses to follow-up. To test the feasibility of recruitment, filming and referral, a convenience sample of 40 neonates was planned for. Based on our previous experience from the hospital, around 2 % of deliveries were expected to fill the inclusion criteria [28].

For peer review only - http://bmjopen.bmj.com/site/about/guidelines.xhtml

Intervention

The NeuroMotion™ app was translated from Swedish to Nepali and English and adapted to the local context by adding pictures and possibility for storing videos on the phone for later submission in case internet was unavailable at the time of filming (Figure 1). A trained research assistant guided the parents for installing the app on their smartphones. Due to delay of the app modification, parents of the first 22 infants were instructed to do the installation at home while nine families received the app before discharge from the hospital and two parents without smartphones were invited to come to the hospital to have their child filmed by a research assistant.

Parents were provided with individual log in credentials to the app. All parents were instructed to film two videos of their child during the fidgety movements (FM) period 12-16 weeks post-term age [9]. The app automatically sent notifications to the parents when their child was 12 weeks post-term age. The first notification was sent 3 days before scheduled filming, second on the day of the filming and third 3 days after the scheduled date if no film had been sent in by then. The research team contacted the parents by phone if no film had been uploaded after the third notification. A second film was requested by a notification in the app two days after the first film had been sent or by phone after the film analysis, whichever came first.

The filming took place at home or at the hospital by positioning the child in a supine position when awake and satisfied with arms and legs visible in the screen according to the GMA guidelines [9]. Duration of the films was 2 to 3 minutes each as in previous studies [17,18]. The app automatically uploaded the films to a secure server at Linköping University, Sweden. Apart from the videos and the pseudonymized study ID, no other data were transferred. All videos were analysed according to Prechtl's GMA by three independent certified evaluators blinded to the medical history of the patient [9]. Results were recorded as present (continuous, intermittent or abnormal), or absent (sporadic or absent) FMs. The assessments were discussed in meetings held every second week. When more than one film was available for a child, the best result category was recorded.

The final consensus agreement was reported to the researchers in Nepal, who in turn informed the families via phone. In case of not approved technical quality or absent FMs, one further recording was requested. Babies with consistently absent FMs were examined by a paediatrician at the Paropakar Maternity and Women's Hospital (PP) and offered referral for early intervention program at Self-help Group for Cerebral Palsy, Lalitpur, after parental consent. The research team offered to cover the costs of the rehabilitation.

After receiving feedback about their child's GMA, parents were interviewed by phone about usability of the app by applying a multiple-choice questionnaire in Nepali. The survey consisted of 19 questions with answers on a five-point Likert scale with an option for free text answer. The questions were based on a survey developed by Kwong et al. for evaluation of the BabyMoves app in Australia [29]. Similar questions have also been used in studies in Europe [17,18].

For peer review only - http://bmjopen.bmj.com/site/about/guidelines.xhtml

Outcomes and statistical analyses

Feasibility was defined as the extent to which smartphone aided remote screening could be successfully carried out within our setting [30]. Following outcomes were measured quantitatively:

a) *Reach* of the screening program was evaluated by proportion of participating children in each stage of the follow-up. Background factors between children to parents who successfully returned at least one film with approved technical quality were compared with those who did not by using independent samples t-test for normally distributed continuous variables and Pearson's chi-square test or Fischer's exact test for categorical variables depending on the cell count. P<0.05 was considered statistically significant. Crude Odds Ratios with 95 % confidence intervals were counted for the categorical variables.

b) The *technical quality* of the videos produced was evaluated by a single evaluator (HEKS) using standardized GMA Trust questionnaire (Supplementary Table 1). Each video was assessed by eight parameters. Each parameter was scored as either "Excellent", "Good" or "Indistinct". Presence of any single parameter with "Indistinct" score made the video "Not approved" for technical quality . Quality of the GMA assessment was evaluated by calculating inter evaluator agreement with Krippendorf alpha for videos with approved technical quality [31].

c) Parental perceptions of the *usability* of the app were evaluated by a multiple-choice questionnaire and the results were presented using stacked bar graphs.

Qualitative evaluation on the acceptability of the intervention will be reported separately (submitted manuscript).

Patient and public involvement

No patient involved.

Ethics

Oral and written information was provided to at least one of the parents and written consent collected prior to enrolment. The research assistant giving the information was hired for the study and not part of the clinical care team. Ethical permit for the study was granted by the Institutional Review Committee of the Paropakar Maternity and Women's Hospital (61/1978) and for data analysis by the Swedish Ethical Review Authority (2020-07168).

Results

Reach and Quality

Rate of birth asphyxia was lower than expected during the study period and more families were ineligible due to residence outside of the study area. Furthermore, during the first half of the study mortality at the NICU/SNCU was higher than expected and more infants were transferred to other hospitals. Therefore only 34 eligible infants were identified and 31 of them enrolled despite

For peer review only - http://bmjopen.bmj.com/site/about/guidelines.xhtml

extension of the recruitment period by a month (Figure 2). Male predominance was notable with only six female infants recruited into the study.

Altogether, 46 videos were received from 20 families. All films were made between 12 and 16 weeks of post-term age and thus suitable for GMA analysis. The technical quality was approved in 35 out of 46 videos and 16 children had at least one video with approved technical quality (Supplementary Table 2). GMA scoring could confidently be performed in four additional videos with not approved technical quality bringing the total number of children assessed by GMA to 18. The most common quality issues leading to rejection of videos were inability to hold the camera still (n=15, 33%), wrong position of camera for filming (n=12, 26%) and too busy background (n=11, 24%). The level of agreement between the GMA evaluators was acceptable (Krippendorf alpha 0.781 that all three assessors agreed).

There were no statistically significant differences in the characteristics between children whose parents succeeded in returning at least one film of approved technical quality versus those who did not (Table 1). Three children had consistently absent FMs. One family moved abroad and could not be reached for follow-up. Two children were evaluated by a paediatrician (PP) and recommended early intervention, but their parents decided against joining the program.

Usability

A response to the usability survey was provided by parents of 25 participating children including 18 parents who had succeeded in filming their child and seven who had attempted to use the app but not succeeded in sending a video (Figure 3). Most of the parents found that the app was easy to use, and instructions were easy to follow. The screen filter outlining the contours of a child helped parents in positioning the camera and sending the videos was easy in most of the cases. More than half of the participants experienced worry about their child’s development when using the app and most felt using the app increased their awareness of their child’s development. A face-to-face assessment would have been preferable to filming to eight respondents (32%).

Discussion

This study examined the feasibility of implementing smartphone aided remote GMA for screening of infants for high risk of CP in Nepal. We found that just short of half of eligible parents (16/34) were able to return at least one film of approved technical quality. Overall, 35 out of 46 films were of approved technical quality. Parental perceptions of the follow-up were positive.

To our knowledge, this is the first time that a smartphone app has been used for assessing GMA outside high-income settings. Our group was more likely to consent to participate (31/34, 91%) than that reported in an Australian cohort of preterm infants (227/273, 83%) [29]. However, the proportion successfully filming was lower in Nepal (16/31, 52%) than in Australia (158/226, 70%) [29], Belgium, Denmark and Norway (69/86, 80%) [17], or Sweden (36/37, 97%) [18]. This was mostly due to technical quality of films being lower than in the previous studies that all reported approved quality in more than 95% of the videos. This contrasts with the findings of the usability

For peer review only - http://bmjopen.bmj.com/site/about/guidelines.xhtml

survey, where vast majority of the Nepalese parents reported that they found using the app easy and instructions easy to follow.

Picture sharpness alone rarely was an issue whereby we infer that to improve the success rate of filming, more emphasis should be put on parental training in future studies. In our study, only nine participants received hands-on assistance in installation of the app at the hospital and the rest were guided remotely by phone. A follow-up by phone at six weeks' age was added to reduce attrition and a telephone contact was made in most cases at the time of filming. Kwong et al. in Australia recently demonstrated that simple instructions enabled parents to successfully film their children at home for centralized GMA [32]. A small pilot study in India did the same using WhatsApp messaging suggesting that a separate app for GMA might not be needed [33]. However, privacy regulations and safety of data transfer and storage need to be considered when implementing telemedicine interventions [15].

Parents participating in the study had positive experience of the NeuroMotion™ app and the follow-up in general. The response rate to the usability survey was higher than in the previous studies and we also interviewed parents who had failed in filming their child as intended. The results were similar to HIC settings with the Nepalese and Australian parents showing higher rates of worry and preference to physical examination than the other two studies [17, 18, 29]. A separate qualitative study will explore the experiences of the participating parents in-depth (manuscript submitted).

The United Nations' Sustainable Development Goals agenda of leaving no child behind [34] can only be achieved if comprehensive neonatal follow-up is provided simultaneously to improvement in pre- and peripartum care. The validity of GMA in LMIC settings is yet to be proven as most studies conducted thus far have been small like ours [12,13]. Broad developmental assessment should be provided to children at risk of disability as the GMA has poorer predictive ability for other adverse neurodevelopmental outcomes than CP [35]. A report from Australia published during the Covid-19 pandemic showed that GMA analysis could be made during a Zoom call as a part of a comprehensive developmental evaluation [36]. In LMIC settings, GMA could be added to risk child follow-up by engaging community health workers in filming, which might improve the quality of the films. In India, an on-going trial is evaluating the possibility of teaching community health workers the GMA method [3], and in future automatized analysis could help in mass screening of videos when filming is done in a standardized manner [19].

Lastly, ensuring linkage to an early intervention program is imperative for ethical conduct of GMA screening. In our study three children at high risk of CP with absent FMs were identified, but one of the families moved abroad and the other two were not willing to enrol in the offered free intervention program. The ability of GMA to identify children at high risk of CP before overt signs of the condition appear can be a drawback in settings like Nepal, where there is no tradition for postnatal follow-up and parents have limited resources available. Furthermore, care seeking from alternative providers is common in Nepal as CP is perceived to be caused by also other than medical reasons [24,37]. Further qualitative studies might shed light to the appropriate ways of

For peer review only - http://bmjopen.bmj.com/site/about/guidelines.xhtml

informing parents about the diagnosis of high risk CP in low- and middle-income settings [38]. Early intervention conducted partially via digital platform might also increase the uptake considering the overall positive attitudes parents showed towards remote screening.

Limitations

This study was a small single-centre feasibility study conducted in the capital city of Nepal with relatively high standard of living, education and mobile phone access and the results are thus not generalizable to rural areas of the country, where remote screening could be most needed. Despite extending recruitment period we did not reach the planned sample size. The study was not powered to properly examine background predictors that might have impacted filming success and it is essential that alternative means of assessment are provided parallel to remote assessment to avoid discriminating disadvantaged groups such as females who only represented 20% of the enrolled infants.

We attempted to minimize participation bias by interviewing parents who did not succeed in filming. However, as the interview was conducted by phone by the same research assistant who had provided guidance regarding the app installation and the results of the GMA analysis, it is possible that social desirability bias influenced parental responses. Leading prompts might have also contributed to the positive results.

Finally, the study did not assess the acceptability of remote GMA from the provider point of view, a necessary consideration along with legislation and infrastructure for future scale-up of the intervention [14,15].

Conclusion

Engaging parents in screening of CP with the help of remote GMA is possible in the urban area of a mountainous South Asian lower middle-income country and generally welcomed by the participating parents.

Contributorship statement:

AJK, AKC, HEKS, HL, JW, KUS, NB, PP and RG designed the study.
AJK, AKC, KUS, OB, PB, PP and RG collected the data and/or supervised data acquisition.
AJK, HEKS, KS and OB analysed the data.
AJK drafted the manuscript and had access to all the data. All authors participated in revising the manuscript critically.
All authors approve the submitted version.

Acknowledgements: Asmita Basnet recruited the study participants and guided the parents in installing the NeuroMotion™ app in the hospital. Rabina Karki conducted the phone follow-up, provided support for the parents during filming and administered the usability survey. Anna Axelin contributed to the design of the study. The authors wish to thank Alicia Spittle and Amanda Kwon for the permission to use the usability survey questionnaire.

For peer review only

Competing interests:

KS and HEKS developed the app NeuroMotion™ with financial support from a private donation through Knut och Alice Wallenbergs Stiftelse. For the purpose of further research and later implementation of NeuroMotion™, the company NeuroMotion AB was founded and is the owner of the app. NeuroMotion AB is owned by KS and HEKS and has the purpose of research and to be nonprofitable.

Funding:

The project was funded by grants from Stiftelsen Promobilia, 21009, Linnéa and Josef Carlssons stiftelse, Stiftelsen Folke Bernadottes Minnesfond and Födelsefonden. Heléne Sundelin is funded by Region Stockholm, clinical postdoctoral appointment, 2019-1138, and a private donation through the Knut och Alice Wallenbergs Stiftelse.

Data sharing statement:

Data available on request from the authors: The data that support the findings of this study are available from the corresponding author upon reasonable request.

Ethics approval:

Institutional Review Committee of the Paropakar Maternity and Women's Hospital (61/1978)
Swedish Ethical Review Authority (2020-07168)

References

1. World Health Organization. Nurturing care for early childhood development: a framework for helping children survive and thrive to transform health and human potential. World Health Organization, United Nations Children's Fund, World Bank Group; 2018.

2. Morgan C, Fetters L, Adde L, Badawi N, Bancale A, Boyd RN, et al. Early Intervention for Children Aged 0 to 2 Years With or at High Risk of Cerebral Palsy: International Clinical Practice Guideline Based on Systematic Reviews. JAMA Pediatr. 2021 Aug 1;175(8):846–58.

3. Toldo M, Varishthananda S, Einspieler C, Tripathi N, Singh A, Verma SK, et al. Enhancing early detection of neurological and developmental disorders and provision of intervention in low-resource settings in Uttar Pradesh, India: study protocol of the G.A.N.E.S.H. programme. BMJ Open. 2020 Nov 1;10(11):e037335.

4. Benfer KA, Novak I, Morgan C, Whittingham K, Khan NZ, Ware RS, et al. Community-based parent-delivered early detection and intervention programme for infants at high risk of cerebral palsy in a low-resource country (Learning through Everyday Activities with Parents (LEAP-CP): protocol for a randomised controlled trial. BMJ Open. 2018 Jun;8(6):e021186.

For peer review only - http://bmjopen.bmj.com/site/about/guidelines.xhtml

5. Nanyunja C, Sadoo S, Kohli-Lynch M, Nalugya R, Nyonyintono J, Muhumuza A, et al. Early care and support for young children with developmental disabilities and their caregivers in Uganda: The Baby Ubuntu feasibility trial. Front Pediatr. 2022 Sep 13;10:981976.

6. McIntyre S, Goldsmith S, Webb A, Ehlinger V, Hollung SJ, McConnell K, et al. Global prevalence of cerebral palsy: A systematic analysis. Developmental Medicine & Child Neurology. 2022;64(12):1494–506.

7. Scherzer AL, Chhagan M, Kauchali S, Susser E. Global perspective on early diagnosis and intervention for children with developmental delays and disabilities. Dev Med Child Neurol. 2012 Dec;54(12):1079–84.

8. Al Imam MH, Jahan I, Muhit M, Hardianto D, Laryea F, Chhetri AB, et al. Predictors of Rehabilitation Service Utilisation among Children with Cerebral Palsy (CP) in Low- and Middle-Income Countries (LMIC): Findings from the Global LMIC CP Register. Brain Sciences. 2021 Jun 25;11(7):848.

9. Einspieler C, Prechtl HFR, Bos AF, Ferrari F, Cioni G. Prechtl's Method on the Qualitative Assessment of General Movements in Preterm, Term and Young Infants. Cambridge University Press; 2005. 104 p.

10. Kwong AKL, Fitzgerald TL, Doyle LW, Cheong JLY, Spittle AJ. Predictive validity of spontaneous early infant movement for later cerebral palsy: a systematic review. Dev Med Child Neurol. 2018;60(5):480–9.

11. Novak I, Morgan C, Adde L, Blackman J, Boyd RN, Brunstrom-Hernandez J, et al. Early, Accurate Diagnosis and Early Intervention in Cerebral Palsy: Advances in Diagnosis and Treatment. JAMA Pediatr. 2017 Sep 1;171(9):897–907.

12. King AR, Al Imam MH, McIntyre S, Morgan C, Khandaker G, Badawi N, et al. Early Diagnosis of Cerebral Palsy in Low- and Middle-Income Countries. Brain Sci. 2022 Apr 23;12(5):539.

13. Tomantschger I, Herrero D, Einspieler C, Hamamura C, Voos MC, Marschik PB. The general movement assessment in non-European low- and middle-income countries. Rev Saude Publica. 2018 Jan 29;52(6):1–8.

14. Fleming KA, Horton S, Wilson ML, Atun R, DeStigter K, Flanigan J, et al. The Lancet Commission on diagnostics: transforming access to diagnostics. The Lancet. 2021;398(10315):1997–2050.

15. World Health Organization. WHO guideline recommendations on digital interventions for health system strengthening. 2019;

For peer review only - http://bmjopen.bmj.com/site/about/guidelines.xhtml

16. Spittle A, Olsen J, Kwong A, Doyle L, Marschik P, Einspieler C, et al. The Baby Moves prospective cohort study protocol: using a smartphone application with the General Movements Assessment to predict neurodevelopmental outcomes at age 2 years for extremely preterm or extremely low birthweight infants. BMJ open. 2016;6(10):e013446.

17. Adde L, Brown A, Van Den Broeck C, Decoen K, Eriksen BH, Fjørtoft T, et al. In-Motion-App for remote General Movement Assessment: A multi-site observational study. BMJ Open. 2021;11(3).

18. Svensson KA, Örtqvist M, Bos AF, Eliasson AC, Sundelin HEK. Usability and inter-rater reliability of the NeuroMotion app: A tool in General Movements Assessments. European Journal of Paediatric Neurology. 2021 Jul;33:29–35.

19. Silva N, Zhang D, Kulvicius T, Gail A, Barreiros C, Lindstaedt S, et al. The future of General Movement Assessment: The role of computer vision and machine learning - A scoping review. Res Dev Disabil. 2021 Mar;110:103854.

20. Vandenbroucke JP, Elm E von, Altman DG, Gøtzsche PC, Mulrow CD, Pocock SJ, et al. Strengthening the Reporting of Observational Studies in Epidemiology (STROBE): Explanation and Elaboration. PLOS Medicine. 2007 Oct 16;4(10):e297.

21. Eldridge SM, Chan CL, Campbell MJ, Bond CM, Hopewell S, Thabane L, et al. CONSORT 2010 statement: extension to randomised pilot and feasibility trials. Pilot Feasibility Stud. 2016;2:64.

22. Lancaster GA, Thabane L. Guidelines for reporting non-randomised pilot and feasibility studies. Pilot and Feasibility Studies. 2019 Oct 6;5(1):114.

23. Ministry of Health and Population, Nepal, New ERA, Kathmandu, ICF. Nepal Demographic and Health Survey 2022: Key Indicators Report. Ministry of Health and Population, Kathmandu, Nepal; 2022. ‘

24. Thapa R. Symptom Recognition and Diagnosis of Cerebral Palsy in Nepal. J Autism Dev Disord. 2017;47(6):1739–48.

25. Banskota B, Shrestha S, Rajbhandari T, Banskota AK, Spiegel DA. A Snapshot of 1001 Children Presenting with Cerebral Palsy to a Children's Disability Hospital. J Nepal Health Res Counc. 2015 Apr;13(29):31–7.

26. World Health Organization Regional Office for South-East Asia. Compilation of community-based rehabilitation practices in the WHO South-East Asia Region; 2013

27. Buderer NM. Statistical methodology: I. Incorporating the prevalence of disease into the sample size calculation for sensitivity and specificity. Acad Emerg Med Off J Soc Acad Emerg Med. 1996 Sep;3(9):895–900.

For peer review only

28. Kc A, Wrammert J, Clark RB, Ewald U, Vitrakoti R, Chaudhary P, et al. Reducing Perinatal Mortality in Nepal Using Helping Babies Breathe. Pediatrics. 2016;137(6).

29. Kwong AK, Eeles AL, Olsen JE, Cheong JL, Doyle LW, Spittle AJ. The Baby Moves smartphone app for General Movements Assessment: Engagement amongst extremely preterm and term-born infants in a state-wide geographical study. J Paediatr Child Health. 2018 Oct 4;

30. Proctor E, Silmere H, Raghavan R, Hovmand P, Aarons G, Bunger A, et al. Outcomes for implementation research: Conceptual distinctions, measurement challenges, and research agenda. Administration and Policy in Mental Health and Mental Health Services Research. 2011;38(2):65–76.

31. Hayes A, Krippendorff K. Answering the Call for a Standard Reliability Measure for Coding Data. Communication Methods and Measures. 2007 Apr 1;1:77–89.

32. Kwong AKL, Eeles AL, Olsen JE, Zannino D, Kariotis T, Spittle AJ. Instructional guides for filming infant movements at home are effective for the General Movements Assessment. Journal of Paediatrics and Child Health. 2021;58(5):796–801.

33. Saini L, Madaan P, Bhagwat C, Einspieler C. Home-videos for neurodevelopmental follow-up of high-risk infants during COVID-19 pandemic: a simple and inexpensive tool. Journal of Tropical Pediatrics. 2021;67(1):fmaa088.

34. United Nations General Assembly. Transforming our world: the 2030 Agenda for Sustainable Development. Resolution 70/1. 2015.

35. Caesar R, Colditz PB, Cioni G, Boyd RN. Clinical tools used in young infants born very preterm to predict motor and cognitive delay (not cerebral palsy): a systematic review. Dev Med Child Neurol. 2021 Apr;63(4):387–95.

36. Maitre NL, Benninger KL, Neel ML, Haase JA, Pietruszewski L, Levengood K, et al. Standardized Neurodevelopmental Surveillance of High-risk Infants Using Telehealth: Implementation Study during COVID-19. Pediatr Qual Saf. 2021 Jul 28;6(4):e439.

37. Saul R, Phillips D. Ghosts and germs: Cerebral Palsy in Nepal: A preliminary exploration of cosmology and disability. Disability and development: Learning from action and research on disability in the majority world. 1999;

38. Byrne R, Duncan A, Pickar T, Burkhardt S, Boyd RN, Neel ML, et al. Comparing parent and provider priorities in discussions of early detection and intervention for infants with and at risk of cerebral palsy. Child: Care, Health and Development. 2019;45(6):799–807.

For peer review only

For peer review only - http://bmjopen.bmj.com/site/about/guidelines.xhtml

Figure and Table Legends

Figure 1: Screenshots of the Nepali version of the NeuroMotion™ app including a) introduction, b-d) instructions about filming, and e) a view of the filming interface.

Figure 2: Participant flow diagram. FM =Fidgety Movements, SNCU/NICU = Sick Newborn Care Unit/Neonatal Intensive Care Unit

Figure 3: Stacked bar graph of parental answers to smartphone app usability survey.

Table 1: Background characteristics. Comparison between those participants who returned at least one film of approved technical quality and those who did not.

For peer review only

For peer review only - http://bmjopen.bmj.com/site/about/guidelines.xhtml

Supporting information

Supplementary Table 1: General Movements trust specialist assessment of the technical quality of the NeuroMotion™ films.

Supplementary Table 2: Results of the technical quality assessment and evaluation of Fidgety Movements.

For peer review only

For peer review only - http://bmjopen.bmj.com/site/about/guidelines.xhtml

Table 1:

	Filmed successfully = 16	Not filmed successfully = 15	p-value	cOR (95%CI)
INFANT CHARACTERISTICS				
Gestational age in weeks, Mean (SD)	38.9 (1.4)	38.8 (1.5)	0.32*	-
Apgar at 5 min, Mean (SD)	5.3 (0.4)	4.8 (0.7)	0.67*	-
Resuscitation at 5 min	16	14	0.29**	NA
Clinical seizures	3	7	0.14***	0.26 (0.05-1.32)
Sex			1.00***	
Female	3	3		Ref
Male	13	12		1.08 (0.18-6.42)
Mode of delivery			0.25***	
Normal vaginal	13	9		Ref
Instrumental	0	0		-
Caesarean section	3	6		0.34 (0.06-1.75)
DEMOGRAPHIC FACTORS				
Maternal education			0.36***	
1-7 years	5	3		Ref
None	0	2		-
Complete primary	3	2		0.45 (0.05-3.57)
Complete secondary	3	6		0.30 (0.04-2.20-
University degree	5	2		1.50 (0.17-13.22)
Maternal ethnicity			0.90**	
Relatively disadvantageous	11	10		Ref
Relatively advantageous	5	5		1.10 (0.24-4.96)
Maternal age, Mean (SD)	25y5m (3y6m)	24y2m (3y)	0.60*	
Paternal ethnicity			0.90**	
Relatively disadvantageous	11	10		Ref
Relatively advantageous	5	5		1.10 (0.24-4.96)
Paternal age, Mean (SD)	29y6m (4y7m)	27y7m (3y3m)	0.37*	

For peer review only - http://bmjopen.bmj.com/site/about/guidelines.xhtml

	Filmed successfully = 16	Not filmed successfully = 15	p-value	cOR (95%CI)
SMARTPHONE USE				
Parents own a smart phone	16	15	NA	-
Years with smart phone, Mean (SD)	9y1m (3y2m)	7y7m (3y)	0.68*	-
Parents use social media	15	15	0.33**	NA
App installation			1.00***	
Hospital	5	4	1.00	Ref
Home	11	11	1.00	0.80 (0.16-3.79)

CI = Confidence Interval, cOR = crude Odds Ratio, SD = Standard Deviation. No missing data. *independent samples t-test, ** Pearson's chi-square test if a cell has count 5 or more, ***Fischer's exact text if a cell has count less than 5. Parental ethnicity was self-reported. Relatively disadvantageous = Dalit, Janajati, Madhesi or Muslim, Relatively advantageous = Brahmini, Chhetri or other.

For peer review only

For peer review only - http://bmjopen.bmj.com/site/about/guidelines.xhtml

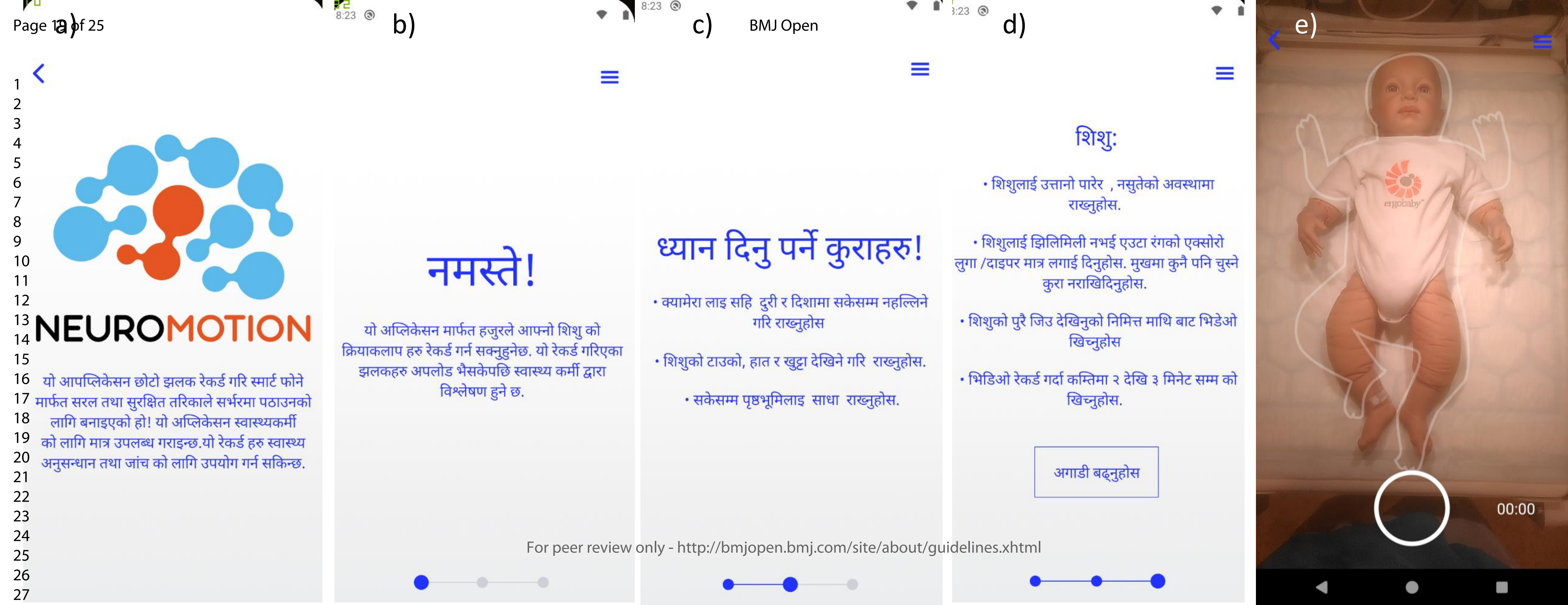
a)
NEUROMOTION
यो आपप्लिकेसन छोटो झलक रेकर्ड गरि स्मार्ट फोने मार्फत सरल तथा सुरक्षित तरिकाले सर्भरमा पठाउनको लागि बनाइएको हो! यो अप्लिकेसन स्वास्थ्यकर्मी को लागि मात्र उपलब्ध गराइन्छ.यो रेकर्ड हरु स्वास्थ्य अनुसन्धान तथा जांच को लागि उपयोग गर्न सकिन्छ.
b)
8:23
नमस्ते!
यो अप्लिकेसन मार्फत हजुरले आफ्नो शिशु को क्रियाकलाप हरु रेकर्ड गर्न सक्नुहुनेछ. यो रेकर्ड गरिएका झलकहरु अपलोड भैसकेपछि स्वास्थ्य कर्मी द्वारा विश्लेषण हुने छ.
c)
8:23
ध्यान दिनु पर्ने कुराहरु!
• क्यामेरा लाइ सहि दुरी र दिशामा सकेसम्म नहल्लिने गरि राख्नुहोस
• शिशुको टाउको, हात र खुट्टा देखिने गरि राख्नुहोस.
• सकेसम्म पृष्ठभूमिलाइ साधा राख्नुहोस.
d)
8:23
शिशु:
• शिशुलाई उत्तानो पारेर , नसुतेको अवस्थामा राख्नुहोस.
• शिशुलाई झिलिमिली नभई एउटा रंगको एक्सोरो लुगा /डाइपर मात्र लगाई दिनुहोस. मुखमा कुनै पनि चुस्ने कुरा नराखिदिनुहोस.
• शिशुको पुरै जिउ देखिनुको निमित्त माथि बाट भिडेओ खिच्नुहोस
• भिडिओ रेकर्ड गर्दा कम्तिमा २ देखि ३ मिनेट सम्म को खिच्नुहोस.
अगाडी बढ्नुहोस
e)
ergobaby
00:00

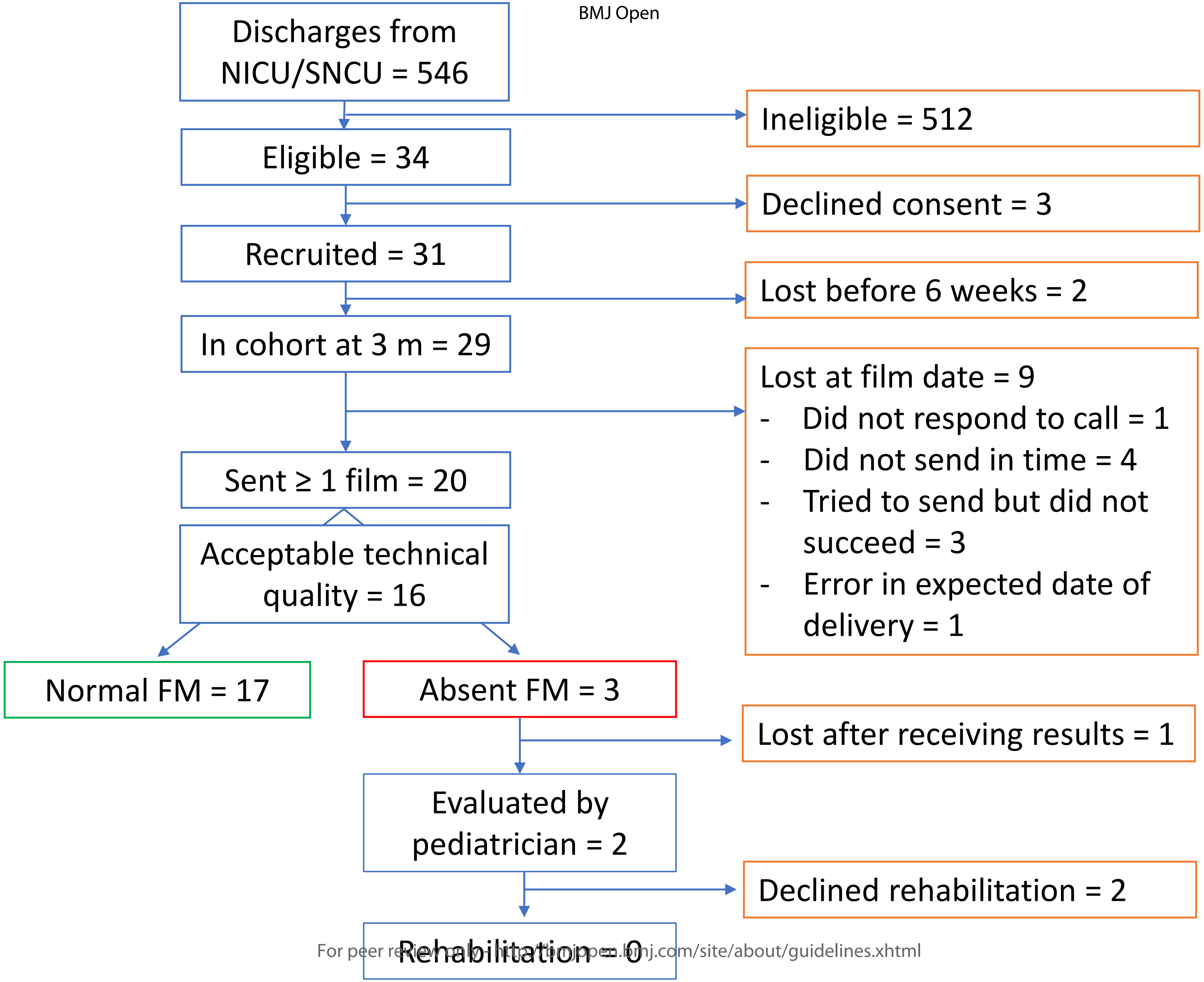
Discharges from NICU/SNCU = 546
Ineligible = 512
Eligible = 34
Declined consent = 3
Recruited = 31
Lost before 6 weeks = 2
In cohort at 3 m = 29
Lost at film date = 9
- Did not respond to call = 1
- Did not send in time = 4
- Tried to send but did not succeed = 3
- Error in expected date of delivery = 1
Sent ≥ 1 film = 20
Acceptable technical quality = 16
Normal FM = 17
Absent FM = 3
Lost after receiving results = 1
Evaluated by pediatrician = 2
Declined rehabilitation = 2
Rehabilitation = 0

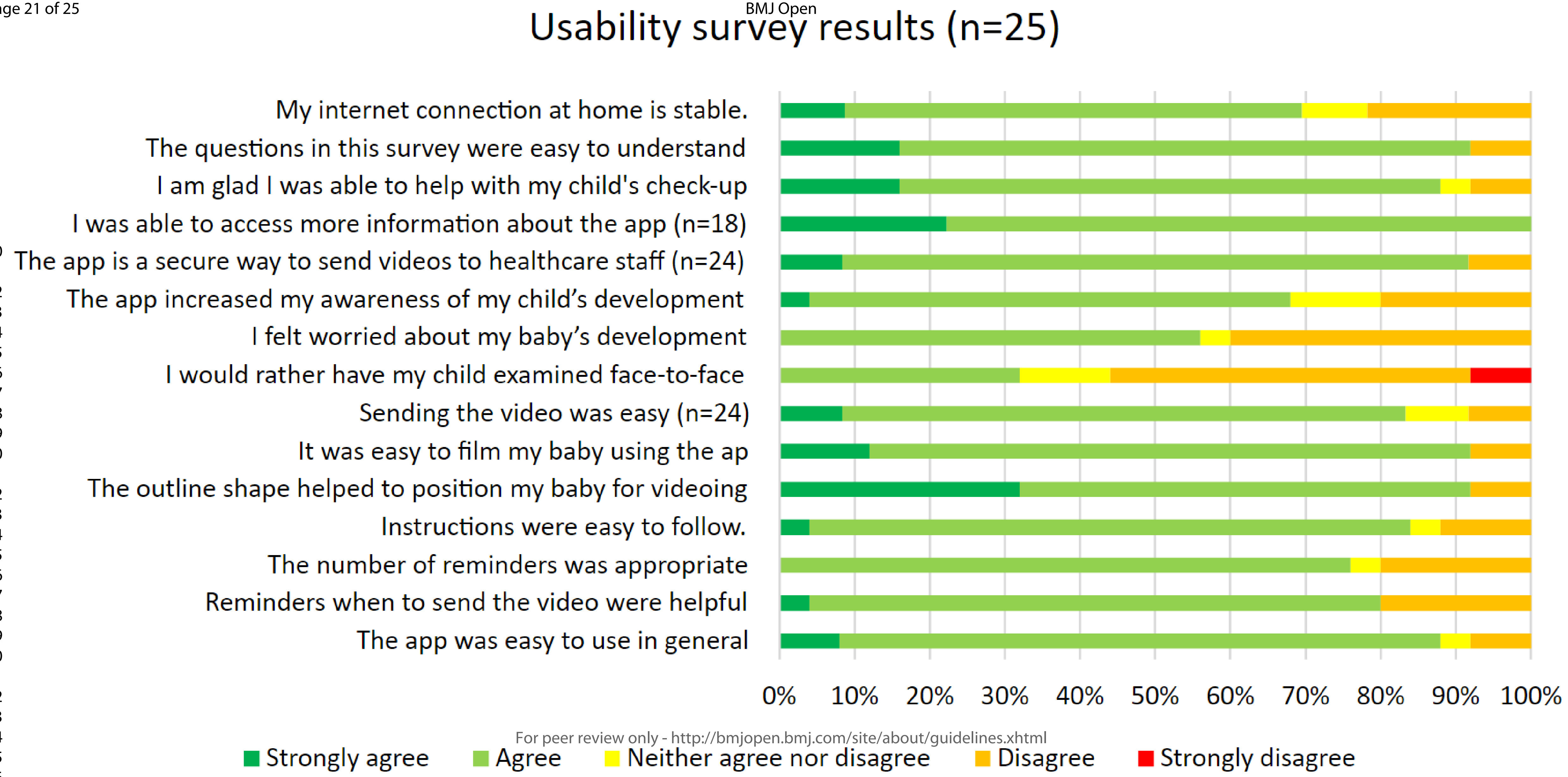
Usability survey results (n=25)
My internet connection at home is stable.
The questions in this survey were easy to understand
I am glad I was able to help with my child's check-up
I was able to access more information about the app (n=18)
The app is a secure way to send videos to healthcare staff (n=24)
The app increased my awareness of my child's development
I felt worried about my baby's development
I would rather have my child examined face-to-face
Sending the video was easy (n=24)
It was easy to film my baby using the ap
The outline shape helped to position my baby for videoing
Instructions were easy to follow.
The number of reminders was appropriate
Reminders when to send the video were helpful
The app was easy to use in general
0%
10%
20%
30%
40%
50%
60%
70%
80%
90%
100%
Strongly agree
Agree
Neither agree nor disagree
Disagree
Strongly disagree

Film #

Assessed by:____

Date:____

Quality	Approved		Not approved	Comments
	Excellent	Good	Indistinct	
Picture quality				
Sharpness				
Camera is held in correct position				
Camera is held in a steady position				
Baby setting				
Clothing				
Background/blanket				
Supine position				
Surrounding influences				
Baby´s mood				
Overall judgement:				

For peer review only

		Picture quality			Baby setting					Overall judgement	Fidgety Movements
Film number	Case number	Sharpness	Camera held in correct pos.	Camera held steady	Clothing	Background/ blanket	Supine position	Surrounding influences	Baby's mood		
1	1	G	E	G	E	E	E	E	E	Approved	F-
2	1	G	I	G	E	E	E	E	E	Approved	F-
3	1	G	G	G	E	E	E	E	E	Approved	F-
4	2	E	I	I	E	G	E	E	E	Approved	F+
5	2	E	I	I	E	E	E	E	E	Rejected	?
6	2	E	I	I	E	G	E	E	E	Approved	F+
7	3	E	E	I	E	G	E	E	E	Approved	F+
8	4	E	E	G	E	G	E	E	E	Approved	F+
9	4	E	E	G	E	G	E	E	E	Approved	F+
10	4	E	E	G	E	G	E	G	E	Approved	F+
11	4	E	E	G	E	G	G	I	E	Rejected	F+
12	4	E	I	I	E	G	G	I	E	Rejected	?
13	4	E	E	E	E	G	E	G	E	Approved	F+
14	5	E	E	E	G	E	E	E	E	Approved	F+
15	5	E	E	E	G	I	E	E	E	Approved	F+
16	5	E	G	E	E	G	E	E	E	Approved	F+
17	5	E	E	E	E	E	E	E	E	Approved	F+
18	5	E	E	E	E	E	E	E	E	Approved	F+
19	6	E	E	I	E	E	E	E	E	Approved	F+
20	6	E	E	G	E	E	E	E	E	Approved	F+
21	7	E	E	G	E	I	E	I	G	Approved	F+
22	7	E	E	I	G	I	E	E	E	Approved	F+
23	8	E	I	I	G	G	E	I	E	Rejected	F+
24	9	G	G	G	E	I	E	G	E	Approved	F+
25	10	E	G	G	E	G	E	G	E	Approved	F-
26	10	G	E	I	E	I	E	I	E	Approved	F-
27	10	E	G	I	E	G	E	G	E	Approved	F-
28	11	E	I	I	I	I	E	E	E	Rejected	?
29	11	E	I	I	E	E	E	I	E	Rejected	F+
30	12	I	E	G	I	G	E	E	E	Rejected	?
31	12	I	G	G	I	I	G	E	E	Rejected	F+
32	12	E	G	E	G	E	E	E	E	Approved	F+
33	12	E	G	G	I	I	E	I	E	Approved	F+
34	13	E	G	G	E	E	E	E	E	Approved	F+
35	13	E	E	G	E	E	E	E	E	Approved	F+
36	14	E	I	I	E	I	E	I	E	Rejected	F-(?)
37	15	E	G	G	E	E	E	E	E	Approved	F+

For peer review only

38	16	E	I	I	I	I	E	I	E	Rejected	?
39	16	E	E	E	G	E	E	E	E	Approved	F+
40	16	E	E	E	G	E	E	E	E	Approved	F+
41	17	E	E	E	E	E	E	E	E	Approved	F+
42	18	E	E	E	E	E	E	E	E	Approved	F+
43	18	E	E	E	E	E	E	G	E	Approved	F+
44	19	E	G	E	E	E	E	E	E	Approved	F+
45	19	E	I	G	E	G	E	E	E	Approved	F+
46	20	E	I	I	E	I	E	E	E	Rejected	F+
Summary of results	Excellent	39	23	13	34	20	43	31	45		
	Good	5	11	18	7	15	3	6	1		
	Indistinct	2	12	15	5	11	0	9	0		
Films number 11, 23, 29 and 31 were rejected for technical quality, but Fidgety Movements were clearly observed.											
Case number 14 only sent one film that was rejected due to poor technical quality, but Fidgety Movements were not observed.											

For peer review only

STROBE Statement—Checklist of items that should be included in reports of ***cohort studies***

	Item No	Recommendation	
**Title and abstract**	1	(*a*) Indicate the study's design with a commonly used term in the title or the abstract	1
		(*b*) Provide in the abstract an informative and balanced summary of what was done and what was found	2
**Introduction**			
Background/rationale	2	Explain the scientific background and rationale for the investigation being reported	3
Objectives	3	State specific objectives, including any prespecified hypotheses	5
**Methods**			
Study design	4	Present key elements of study design early in the paper	3
Setting	5	Describe the setting, locations, and relevant dates, including periods of recruitment, exposure, follow-up, and data collection	3-5
Participants	6	(*a*) Give the eligibility criteria, and the sources and methods of selection of participants. Describe methods of follow-up	4
		(*b*) For matched studies, give matching criteria and number of exposed and unexposed	N/A
Variables	7	Clearly define all outcomes, exposures, predictors, potential confounders, and effect modifiers. Give diagnostic criteria, if applicable	5
Data sources/ measurement	8*	For each variable of interest, give sources of data and details of methods of assessment (measurement). Describe comparability of assessment methods if there is more than one group	4,5
Bias	9	Describe any efforts to address potential sources of bias	8
Study size	10	Explain how the study size was arrived at	4
Quantitative variables	11	Explain how quantitative variables were handled in the analyses. If applicable, describe which groupings were chosen and why	5, table 1
Statistical methods	12	(*a*) Describe all statistical methods, including those used to control for confounding	5
		(*b*) Describe any methods used to examine subgroups and interactions	N/A
		(*c*) Explain how missing data were addressed	N/A
		(*d*) If applicable, explain how loss to follow-up was addressed	not relevant
		(*e*) Describe any sensitivity analyses	N/A
**Results**			
Participants	13*	(a) Report numbers of individuals at each stage of study—eg numbers potentially eligible, examined for eligibility, confirmed eligible, included in the study, completing follow-up, and analysed	Fig 1
		(b) Give reasons for non-participation at each stage	Fig 1
		(c) Consider use of a flow diagram	Fig 1
Descriptive data	14*	(a) Give characteristics of study participants (eg demographic, clinical, social) and information on exposures and potential confounders	Table 1
		(b) Indicate number of participants with missing data for each variable of interest	table 1
		(c) Summarise follow-up time (eg, average and total amount) N/A	ingress
Outcome data	15*	Report numbers of outcome events or summary measures over time	
Main results	16	(*a*) Give unadjusted estimates and, if applicable, confounder-adjusted estimates and their precision (eg, 95% confidence interval). Make clear which confounders were adjusted for and why they were included only cOR reported	
		(*b*) Report category boundaries when continuous variables were categorized	not relevant
		(*c*) If relevant, consider translating estimates of relative risk into absolute risk for a meaningful time period	

For peer review only

Other analyses	17	Report other analyses done—eg analyses of subgroups and interactions, and sensitivity analyses	N/A
**Discussion**			
Key results	18	Summarise key results with reference to study objectives	7
Limitations	19	Discuss limitations of the study, taking into account sources of potential bias or imprecision. Discuss both direction and magnitude of any potential bias	8
Interpretation	20	Give a cautious overall interpretation of results considering objectives, limitations, multiplicity of analyses, results from similar studies, and other relevant evidence	7
Generalisability	21	Discuss the generalisability (external validity) of the study results	8
**Other information**			
Funding	22	Give the source of funding and the role of the funders for the present study and, if applicable, for the original study on which the present article is based	12

*Give information separately for exposed and unexposed groups.

Note: An Explanation and Elaboration article discusses each checklist item and gives methodological background and published examples of transparent reporting. The STROBE checklist is best used in conjunction with this article (freely available on the Web sites of PLoS Medicine at http://www.plosmedicine.org/, Annals of Internal Medicine at http://www.annals.org/, and Epidemiology at http://www.epidem.com/). Information on the STROBE Initiative is available at http://www.strobe-statement.org.

For peer review only

BMJ Open

NeuroMotion™ Smartphone Application for Remote General Movements Assessment: a Feasibility Study in Nepal

Journal:	*BMJ Open*
Manuscript ID	bmjopen-2023-080063.R2
Article Type:	Original research
Date Submitted by the Author:	31-Jan-2024
Complete List of Authors:	Kukka, Antti; Uppsala University, Department of Women's and Children's Health; Region Gävleborg, Department of Pediatrics<br>Sundelin, Heléne; Linköping University, Department of Biomedical and Clinical Sciences; Karolinska Institutet, Department of Women's and Children's Health<br>Basnet, Omkar; Golden Community<br>Paudel, Prajwal; Paropakar Maternity and Women's Hospital<br>Subedi, Kalpana; Paropakar Maternity and Women's Hospital<br>Svensson, Katarina; Linköping University, Division of Children's and Women's Health, Department of Biomedical and Clinical Sciences<br>brown, nick; Uppsala Universitet, Department of Women's and Children's Health; Region Gävleborg, Department of Pediatrics<br>Litorp, Helena; Uppsala University, Department of Women's and Children's Health; Karolinska Institutet, Department of Global Public Health<br>Gurung, Rejina; Uppsala University, Department of Women's and Children's Health; Golden Community<br>Bhattarai, Pratiksha; Golden Community<br>Wrammert, Johan; Uppsala University, Department of Women's and Children's Health<br>Ashish, KC; Uppsala University, Department of Women's and Children's Health; University of Gothenburg, School of Public Health and Community Medicine
**Primary Subject Heading**:	Paediatrics
Secondary Subject Heading:	Neurology, Health informatics, Global health, Communication, Diagnostics
Keywords:	Developmental neurology & neurodisability < PAEDIATRICS, Feasibility Studies, Nepal, Follow-Up Studies, NEONATOLOGY, eHealth

SCHOLARONE™ Manuscripts

For peer review only - http://bmjopen.bmj.com/site/about/guidelines.xhtml

I, the Submitting Author has the right to grant and does grant on behalf of all authors of the Work (as defined in the below author licence), an exclusive licence and/or a non-exclusive licence for contributions from authors who are: i) UK Crown employees; ii) where BMJ has agreed a CC-BY licence shall apply, and/or iii) in accordance with the terms applicable for US Federal Government officers or employees acting as part of their official duties; on a worldwide, perpetual, irrevocable, royalty-free basis to BMJ Publishing Group Ltd ("BMJ") its licensees and where the relevant Journal is co-owned by BMJ to the co-owners of the Journal, to publish the Work in this journal and any other BMJ products and to exploit all rights, as set out in our licence.

The Submitting Author accepts and understands that any supply made under these terms is made by BMJ to the Submitting Author unless you are acting as an employee on behalf of your employer or a postgraduate student of an affiliated institution which is paying any applicable article publishing charge ("APC") for Open Access articles. Where the Submitting Author wishes to make the Work available on an Open Access basis (and intends to pay the relevant APC), the terms of reuse of such Open Access shall be governed by a Creative Commons licence – details of these licences and which Creative Commons licence will apply to this Work are set out in our licence referred to above.

Other than as permitted in any relevant BMJ Author's Self Archiving Policies, I confirm this Work has not been accepted for publication elsewhere, is not being considered for publication elsewhere and does not duplicate material already published. I confirm all authors consent to publication of this Work and authorise the granting of this licence.

For peer review only

NeuroMotion™ Smartphone Application for Remote General Movements Assessment: a Feasibility Study in Nepal

Author list:

Antti J. Kukka[1,2], Heléne E.K. Sundelin[3,4], Omkar Basnet[5], Prajwal Paudel[6], Kalpana Upadhyaya Subedi[6], Katarina Svensson[3,7], Nick J.W. Brown[1,2], Helena Litorp[1,8], Rejina Gurung[1,5], Pratiksha Bhattarai[5], Johan Wrammert[1] and Ashish KC[1,9]

Affiliations:

1. Department of Women's and Children's Health, Uppsala University, Sweden
2. Department of Pediatrics, Gävle Regional Hospital, Region Gävleborg, Sweden
3. Division of Children´s and Women´s Health, Department of Biomedical and Clinical Sciences, Linköping University, Linköping, Sweden.
4. Neuropediatric Unit, Department of Women's and Children's Health, Karolinska University Hospital, Karolinska Institutet, Stockholm, Sweden
5. Golden Community, Lalitpur, Nepal
6. Paropakar Maternity and Women's Hospital, Kathmandu, Nepal
7. Crown Princess Victoria's Children's and Youth Hospital, University Hospital, Linkoping, Sweden
8. Department of Global Public Health, Karolinska Institutet, Stockholm, Sweden
9. School of Public Health and Community Medicine, University of Gothenburg, Gothenburg, Sweden

Corresponding author: Antti Kukka, Department of Women's and Children's Health, Uppsala University, SE-751 85, Uppsala, Sweden, https://www.katalog.uu.se/profile/?id=N19-2319
Email address: antti.kukka@uu.se

ORCID:

Antti Juhani Kukka https://orcid.org/0000-0002-5879-7417
Heléne Sundelin https://orcid.org/0000-0002-8189-4098
Omkar Basnet https://orcid.org/0000-0001-7328-8189
Prajwal Paudel
Kalpana Subedi https://orcid.org/0000-0002-2019-1997
Katarina Svensson https://orcid.org/0000-0001-9368-8366
Nick Brown https://orcid.org/0000-0003-1789-0436
Helena Litorp https://orcid.org/0000-0001-7164-598X
Rejina Gurung 0000-0002-4262-3543
Pratiksha Bhattarai https://orcid.org/0000-0001-9455-4394
Johan Wrammert orcid.org/0000-0002-9430-7681
Ashish KC https://orcid.org/0000-0002-0541-4486

For peer review only

Abbreviations:

CP = cerebral palsy
FM = fidgety movements
GMA = General Movements Assessment
HIC = high-income country
NICU/SNCU = neonatal intensive care unit/sick newborn care unit

Abstract:

OBJECTIVES: To evaluate the feasibility of using the NeuroMotion™ smartphone application for remote General Movements Assessment for screening of infants for cerebral palsy in Kathmandu, Nepal.

METHOD: Thirty-one term born infants at risk of cerebral palsy due to birth asphyxia or neonatal seizures were recruited for the follow-up at Paropakar Maternity and Women's Hospital, 1st October 2021 to 7th January 2022. Parents filmed their children at home using the app at 3 months' age and the videos were assessed for technical quality using a standardized form and for fidgety movements by Prechtl's General Movements Assessment. Usability of the app was evaluated through a parental survey.

RESULTS: Twenty families sent in altogether 46 videos out of which 35 had approved technical quality. Sixteen children had at least one video with approved technical quality. Three infants lacked fidgety movements. The level of agreement between assessors was acceptable (Krippendorf alpha 0.781). Parental answers to the usability survey were in general positive.

INTERPRETATION: Engaging parents in screening of cerebral palsy with the help of a smartphone-aided remote General Movements Assessment is possible in the urban area of a South Asian lower middle-income country.

Short title: Screening of CP using remote GMA in Nepal

Word count: 3137

Keywords: cerebral palsy, general movements assessment, neonatal follow-up, low- and middle-income countries, pilot projects

Article summary

Strengths and limitations of this study:

- Three independent assessors judged the presence of General Movements in each video.
- Standardized assessment of the technical quality of videos was performed.
- Social desirability bias might have contributed to the positive results in the usability survey.
- Small sample from a single referral hospital limits generalizability of the findings.

For peer review only

Background

All children with disability have the right reach their full developmental potential [1]. Cerebral palsy (CP) is the most common motor disability of childhood and international guidelines recommend early intervention for all children at high risk of CP [2]. A number of on-going trials are evaluating ways to adapt early intervention to low- and middle-income countries (LMICs) [3-5], which shoulder the vast majority of children affected by CP globally [6]. Delay in accessing rehabilitation is common due to poor availability and lack of targeted screening programs for high risk children [7-8].

Affordable and accurate tools are therefore needed to identify children at high risk of CP in LMICs. General Movements Assessment (GMA) is a cheap, non-invasive method for recognizing these children based on observing their spontaneous movements [9]. It has been shown to have excellent validity at 3 months' age and its use in screening of high risk children is now globally endorsed [10-11], although its accuracy in LMIC populations is yet to be established [12]. Lack of providers trained in the methodology has thus far limited its implementation [13], and telemedicine is one way of overcoming this barrier [14,15]. Lockdowns imposed during the recent Covid-19 pandemic added urgency for finding alternatives for traditional face-to-face check-ups.

Previous studies in high-income countries (HICs) have shown that remote GMA using smartphone applications (apps) is feasible [16-18]. Automatized movement analysis using machine learning holds further promise for wider availability [19]. However, to our knowledge no previous study has utilized smartphone apps for remote GMA in LMIC settings, where the disease burden is the greatest. We therefore aimed to evaluate the feasibility of using smartphone aided remote GMA for screening of infants at risk of CP in Kathmandu, the capital of Nepal, a lower middle-income country. Feasibility was assessed through following specific objectives:

1) to estimate the *reach* of the screening program

2) to evaluate the *technical quality* of the videos and *quality of the assessment*

3) to survey parental perceptions of the *usability* of the app.

Methods

A combination of the STROBE checklist for cohort studies [20] and CONSORT statement for pilot and feasibility trials [21] was used for reporting the study following Guidelines for reporting non-randomised pilot and feasibility studies [22].

Study design and setting

This is a single-centre feasibility study conducted at the Paropakar Maternity and Women's Hospital, Kathmandu, Nepal. Recruitment of infants ran from 1st October 2021 to 7th January 2022 just before the onset of third peak of the Covid-19 pandemic and last response to the usability

For peer review only

For peer review only - http://bmjopen.bmj.com/site/about/guidelines.xhtml

survey was registered on 2nd March 2023. Paropakar is a public tertiary hospital acting as the referral hospital for the surrounding Bagmati province. Infants admitted to the hospital's Neonatal Intensive Care Unit/Sick Newborn Care Unit (NICU/SNCU) are offered post-discharge follow-up up at the paediatric outpatient department up to 14 years' age, but few families choose to participate.

Nationally only 70 % of infants receive a post-natal check-up in Nepal [23].Targeted developmental clinics for high risk children are generally lacking whereby delay in diagnosis until nearly school age is common [24,25]. Paediatric rehabilitation services are for CP are provided by non-governmental organizations and government hospitals working by Community Based Rehabilitation principles [26]. Ownership of smartphones in the Bagmati province is 75 and 84 % among women and men respectively [23].

Participants

Term born neonates (≥ 37 completed gestational weeks according to last menstrual period) at risk of CP due to birth asphyxia or seizures were recruited to the follow-up. The included infants had to survive to discharge and fulfil at least one of the following criteria: 1) Apgar score ≤ 5 at 5 minutes; 2) bag-and-mask ventilation ≥ 5 minutes after delivery; or 3) diagnosis of clinical seizures at the NICU/SNCU.

Newborn infants who were transferred to other hospitals, and those whose parents lived outside of the Kathmandu valley with travel time to the hospital above one hour were excluded due to limited availability of rehabilitation resources outside the capital.

All infants potentially eligible for the study were routinely admitted to the NICU/SNCU, which was confirmed by a chart review of 3 preceding months. Paediatricians working at the wards identified eligible infants and informed a research assistant in charge of recruitment. Background demographic data and delivery details were collected by a short parental interview and patient chart review respectively. All participating parents were contacted by a phone call when their child was six weeks old to enquire about the health of the child and to give parents a possibility to ask further questions about the study.

Sample size

In preparation for this study, we calculated that to show the sensitivity and specificity of GMA for CP diagnosis at two years of age with 95% certainty when the expected sensitivity and specificity are 95% and the expected proportion of infants with abnormal outcome in the population is 20%, then using Buderer's formula [27], 365 infants would need to be recruited. A final sample size of 400 would have been needed to account for losses to follow-up. To test the feasibility of recruitment, filming and referral, a convenience sample of 40 neonates was planned for. Based on our previous experience from the hospital, around 2 % of deliveries were expected to fill the inclusion criteria [28].

For peer review only - http://bmjopen.bmj.com/site/about/guidelines.xhtml

Intervention

The NeuroMotion™ app was translated from Swedish to Nepali and English and adapted to the local context by adding pictures and possibility for storing videos on the phone for later submission in case internet was unavailable at the time of filming (Figure 1). A trained research assistant guided the parents for installing the app on their smartphones. Due to delay of the app modification, parents of the first 22 infants were instructed to do the installation at home while nine families received the app before discharge from the hospital and two parents without smartphones were invited to come to the hospital to have their child filmed by a research assistant.

Parents were provided with individual log in credentials to the app. All parents were instructed to film two videos of their child during the fidgety movements (FM) period 12-16 weeks post-term age [9]. The app automatically sent notifications to the parents when their child was 12 weeks post-term age. The first notification was sent 3 days before scheduled filming, second on the day of the filming and third 3 days after the scheduled date if no film had been sent in by then. The research team contacted the parents by phone if no film had been uploaded after the third notification. A second film was requested by a notification in the app two days after the first film had been sent or by phone after the film analysis, whichever came first.

The filming took place at home or at the hospital by positioning the child in a supine position when awake and satisfied with arms and legs visible in the screen according to the GMA guidelines [9]. Duration of the films was 2 to 3 minutes each as in previous studies [17,18]. The app automatically uploaded the films to a secure server at Linköping University, Sweden. Apart from the videos and the pseudonymized study ID, no other data were transferred. All videos were analysed according to Prechtl's GMA by three independent certified evaluators blinded to the medical history of the patient [9]. Results were recorded as present (continuous, intermittent or abnormal), or absent (sporadic or absent) FMs. The assessments were discussed in meetings held every second week. When more than one film was available for a child, the best result category was recorded.

The final consensus agreement was reported to the researchers in Nepal, who in turn informed the families via phone. In case of not approved technical quality or absent FMs, one further recording was requested. Babies with consistently absent FMs were examined by a paediatrician at the Paropakar Maternity and Women's Hospital (PP) and offered referral for early intervention program at Self-help Group for Cerebral Palsy, Lalitpur, after parental consent. The research team offered to cover the costs of the rehabilitation.

After receiving feedback about their child's GMA, parents were interviewed by phone about usability of the app by applying a multiple-choice questionnaire in Nepali. The survey consisted of 19 questions with answers on a five-point Likert scale with an option for free text answer. The questions were based on a survey developed by Kwong et al. for evaluation of the BabyMoves app in Australia [29]. Similar questions have also been used in studies in Europe [17,18].

For peer review only - http://bmjopen.bmj.com/site/about/guidelines.xhtml

Outcomes and statistical analyses

Feasibility was defined as the extent to which smartphone aided remote screening could be successfully carried out within our setting [30]. Following outcomes were measured quantitatively:

a) *Reach* of the screening program was evaluated by proportion of participating children in each stage of the follow-up. Background factors between children to parents who successfully returned at least one film with approved technical quality were compared with those who did not by using independent samples t-test for normally distributed continuous variables and Pearson's chi-square test or Fischer's exact test for categorical variables depending on the cell count. $P<0.05$ was considered statistically significant. Crude Odds Ratios with 95 % confidence intervals were counted for the categorical variables.

b) The *technical quality* of the videos produced was evaluated by a single evaluator (HEKS) using standardized GMA Trust questionnaire (Supplementary Table 1). Each video was assessed by eight parameters. Each parameter was scored as either "Excellent", "Good" or "Indistinct". Presence of any single parameter with "Indistinct" score made the video "Not approved" for technical quality. Quality of the GMA assessment was evaluated by calculating inter evaluator agreement with Krippendorf alpha for videos with approved technical quality [31].

c) Parental perceptions of the *usability* of the app were evaluated by a multiple-choice questionnaire and the results were presented using stacked bar graphs.

Qualitative evaluation on the acceptability of the intervention will be reported separately (submitted manuscript).

Patient and public involvement

No patient involved.

Ethics

Oral and written information was provided to at least one of the parents and written consent collected prior to enrolment. The research assistant giving the information was hired for the study and not part of the clinical care team. Ethical permit for the study was granted by the Institutional Review Committee of the Paropakar Maternity and Women's Hospital (61/1978) and for data analysis by the Swedish Ethical Review Authority (2020-07168).

Results

Reach and Quality

Rate of birth asphyxia was lower than expected during the study period and more families were ineligible due to residence outside of the study area. Furthermore, during the first half of the study mortality at the NICU/SNCU was higher than expected and more infants were transferred to other hospitals. Therefore only 34 eligible infants were identified and 31 of them enrolled despite

For peer review only - http://bmjopen.bmj.com/site/about/guidelines.xhtml

extension of the recruitment period by a month (Figure 2). Male predominance was notable with only six female infants recruited into the study.

Altogether, 46 videos were received from 20 families. All films were made between 12 and 16 weeks of post-term age and thus suitable for GMA analysis. The technical quality was approved in 35 out of 46 videos and 16 children had at least one video with approved technical quality (Supplementary Table 2). GMA scoring could confidently be performed in four additional videos with not approved technical quality bringing the total number of children assessed by GMA to 18. The most common quality issues leading to rejection of videos were inability to hold the camera still (n=15, 33%), wrong position of camera for filming (n=12, 26%) and too busy background (n=11, 24%). The level of agreement between the GMA evaluators was acceptable (Krippendorf alpha 0.781 that all three assessors agreed).

There were no statistically significant differences in the characteristics between children whose parents succeeded in returning at least one film of approved technical quality versus those who did not (Table 1). Three children had consistently absent FMs. One family moved abroad and could not be reached for follow-up. Two children were evaluated by a paediatrician (PP) and recommended early intervention, but their parents decided against joining the program.

Usability

A response to the usability survey was provided by parents of 25 participating children including 18 parents who had succeeded in filming their child and seven who had attempted to use the app but not succeeded in sending a video (Figure 3). Most of the parents found that the app was easy to use, and instructions were easy to follow. The screen filter outlining the contours of a child helped parents in positioning the camera and sending the videos was easy in most of the cases. More than half of the participants experienced worry about their child's development when using the app and most felt using the app increased their awareness of their child's development. A face-to-face assessment would have been preferable to filming to eight respondents (32%).

Discussion

This study examined the feasibility of implementing smartphone aided remote GMA for screening of infants for high risk of CP in Nepal. We found that just short of half of eligible parents (16/34) were able to return at least one film of approved technical quality. Overall, 35 out of 46 films were of approved technical quality. Parental perceptions of the follow-up were positive.

To our knowledge, this is the first time that a smartphone app has been used for assessing GMA outside high-income settings. Our group was more likely to consent to participate (31/34, 91%) than that reported in an Australian cohort of preterm infants (227/273, 83%) [29]. However, the proportion successfully filming was lower in Nepal (16/31, 52%) than in Australia (158/226, 70%) [29], Belgium, Denmark and Norway (69/86, 80%) [17], or Sweden (36/37, 97%) [18]. This was mostly due to technical quality of films being lower than in the previous studies that all reported approved quality in more than 95% of the videos. This contrasts with the findings of the usability

For peer review only - http://bmjopen.bmj.com/site/about/guidelines.xhtml

survey, where vast majority of the Nepalese parents reported that they found using the app easy and instructions easy to follow.

Picture sharpness alone rarely was an issue whereby we infer that to improve the success rate of filming, more emphasis should be put on parental training in future studies. In our study, only nine participants received hands-on assistance in installation of the app at the hospital and the rest were guided remotely by phone. A follow-up by phone at six weeks' age was added to reduce attrition and a telephone contact was made in most cases at the time of filming. Kwong et al. in Australia and a small pilot study in India recently demonstrated that simple instructions for filming could be used for achieving good results with remote GMA where no dedicated app is available [32,33]. However, video file transfer methods would need to comply with local data security and transfer laws [15].

Parents participating in the study had positive experience of the NeuroMotion™ app and the follow-up in general. The response rate to the usability survey was higher than in the previous studies and we also interviewed parents who had failed in filming their child as intended. The results were similar to HIC settings with the Nepalese and Australian parents showing higher rates of worry and preference to physical examination than the other two studies [17, 18, 29]. A separate qualitative study will explore the experiences of the participating parents in-depth (manuscript submitted).

The United Nations' Sustainable Development Goals agenda of leaving no child behind [34] can only be achieved if comprehensive neonatal follow-up is provided simultaneously to improvement in pre- and peripartum care. The validity of GMA in LMIC settings is yet to be proven as most studies conducted thus far have been small like ours [12,13]. Broad developmental assessment should be provided to children at risk of disability as the GMA has poorer predictive ability for other adverse neurodevelopmental outcomes than CP [35]. A report from Australia published during the Covid-19 pandemic showed that GMA analysis could be made during a Zoom call as a part of a comprehensive developmental evaluation [36]. In LMIC settings, GMA could be added to risk child follow-up by engaging community health workers in filming, which might improve the quality of the films. In India, an on-going trial is evaluating the possibility of teaching community health workers the GMA method [3], and in future automatized analysis could help in mass screening of videos when filming is done in a standardized manner [19].

Lastly, ensuring linkage to an early intervention program is imperative for ethical conduct of GMA screening. In our study three children at high risk of CP with absent FMs were identified, but one of the families moved abroad and the other two were not willing to enrol in the offered free intervention program. The ability of GMA to identify children at high risk of CP before overt signs of the condition appear can be a drawback in settings like Nepal, where there is no tradition for postnatal follow-up and parents have limited resources available. Furthermore, care seeking from alternative providers is common in Nepal as CP is perceived to be caused by also other than medical reasons [24,37]. Further qualitative studies might shed light to the appropriate ways of informing parents about the diagnosis of high risk CP in low- and middle-income settings [38].

For peer review only - http://bmjopen.bmj.com/site/about/guidelines.xhtml

Early intervention conducted partially via digital platform might also increase the uptake considering the overall positive attitudes parents showed towards remote screening.

Limitations

This study was a small single-centre feasibility study conducted in the capital city of Nepal with relatively high standard of living, education and mobile phone access and the results are thus not generalizable to rural areas of the country, where remote screening could be most needed. Despite extending recruitment period we did not reach the planned sample size. The study was not powered to properly examine background predictors that might have impacted filming success and it is essential that alternative means of assessment are provided parallel to remote assessment to avoid discriminating disadvantaged groups such as females who only represented 20% of the enrolled infants.

We attempted to minimize participation bias by interviewing parents who did not succeed in filming. However, as the interview was conducted by phone by the same research assistant who had provided guidance regarding the app installation and the results of the GMA analysis, it is possible that social desirability bias influenced parental responses. Leading prompts might have also contributed to the positive results.

Finally, the study did not assess the acceptability of remote GMA from the provider point of view, a necessary consideration along with legislation and infrastructure for future scale-up of the intervention [14,15].

Conclusion

Engaging parents in screening of CP with the help of remote GMA is possible in the urban area of a mountainous South Asian lower middle-income country and generally welcomed by the participating parents.

Contributorship statement:

AJK, AKC, HEKS, HL, JW, KUS, NB, PP and RG designed the study.
AJK, AKC, KUS, OB, PB, PP and RG collected the data and/or supervised data acquisition.
AJK, HEKS, KS and OB analysed the data.
AJK drafted the manuscript and had access to all the data. All authors participated in revising the manuscript critically.
All authors approve the submitted version.

Acknowledgements: Asmita Basnet recruited the study participants and guided the parents in installing the NeuroMotion™ app in the hospital. Rabina Karki conducted the phone follow-up, provided support for the parents during filming and administered the usability survey. Anna Axelin contributed to the design of the study. The authors wish to thank Alicia Spittle and Amanda Kwon for the permission to use the usability survey questionnaire.

For peer review only - http://bmjopen.bmj.com/site/about/guidelines.xhtml

For peer review only

Competing interests:

KS and HEKS developed the app NeuroMotion™ with financial support from a private donation through Knut och Alice Wallenbergs Stiftelse. For the purpose of further research and later implementation of NeuroMotion™, the company NeuroMotion AB was founded and is the owner of the app. NeuroMotion AB is owned by KS and HEKS and has the purpose of research and to be nonprofitable.

Funding:

The project was funded by grants from Stiftelsen Promobilia, 21009, Linnéa and Josef Carlssons stiftelse, Stiftelsen Folke Bernadottes Minnesfond and Födelsefonden. Heléne Sundelin is funded by Region Stockholm, clinical postdoctoral appointment, 2019-1138, and a private donation through the Knut och Alice Wallenbergs Stiftelse.

Data sharing statement:

Data available on request from the authors: The data that support the findings of this study are available from the corresponding author upon reasonable request.

Ethics approval:

Institutional Review Committee of the Paropakar Maternity and Women's Hospital (61/1978)
Swedish Ethical Review Authority (2020-07168)

References

1. World Health Organization. Nurturing care for early childhood development: a framework for helping children survive and thrive to transform health and human potential. World Health Organization, United Nations Children's Fund, World Bank Group; 2018.

2. Morgan C, Fetters L, Adde L, Badawi N, Bancale A, Boyd RN, et al. Early Intervention for Children Aged 0 to 2 Years With or at High Risk of Cerebral Palsy: International Clinical Practice Guideline Based on Systematic Reviews. JAMA Pediatr. 2021 Aug 1;175(8):846–58.

3. Toldo M, Varishthananda S, Einspieler C, Tripathi N, Singh A, Verma SK, et al. Enhancing early detection of neurological and developmental disorders and provision of intervention in low-resource settings in Uttar Pradesh, India: study protocol of the G.A.N.E.S.H. programme. BMJ Open. 2020 Nov 1;10(11):e037335.

4. Benfer KA, Novak I, Morgan C, Whittingham K, Khan NZ, Ware RS, et al. Community-based parent-delivered early detection and intervention programme for infants at high risk of cerebral palsy in a low-resource country (Learning through Everyday Activities with Parents (LEAP-CP): protocol for a randomised controlled trial. BMJ Open. 2018 Jun;8(6):e021186.

For peer review only - http://bmjopen.bmj.com/site/about/guidelines.xhtml

5. Nanyunja C, Sadoo S, Kohli-Lynch M, Nalugya R, Nyonyintono J, Muhumuza A, et al. Early care and support for young children with developmental disabilities and their caregivers in Uganda: The Baby Ubuntu feasibility trial. Front Pediatr. 2022 Sep 13;10:981976.

6. McIntyre S, Goldsmith S, Webb A, Ehlinger V, Hollung SJ, McConnell K, et al. Global prevalence of cerebral palsy: A systematic analysis. Developmental Medicine & Child Neurology. 2022;64(12):1494–506.

7. Scherzer AL, Chhagan M, Kauchali S, Susser E. Global perspective on early diagnosis and intervention for children with developmental delays and disabilities. Dev Med Child Neurol. 2012 Dec;54(12):1079–84.

8. Al Imam MH, Jahan I, Muhit M, Hardianto D, Laryea F, Chhetri AB, et al. Predictors of Rehabilitation Service Utilisation among Children with Cerebral Palsy (CP) in Low- and Middle-Income Countries (LMIC): Findings from the Global LMIC CP Register. Brain Sciences. 2021 Jun 25;11(7):848.

9. Einspieler C, Prechtl HFR, Bos AF, Ferrari F, Cioni G. Prechtl's Method on the Qualitative Assessment of General Movements in Preterm, Term and Young Infants. Cambridge University Press; 2005. 104 p.

10. Kwong AKL, Fitzgerald TL, Doyle LW, Cheong JLY, Spittle AJ. Predictive validity of spontaneous early infant movement for later cerebral palsy: a systematic review. Dev Med Child Neurol. 2018;60(5):480–9.

11. Novak I, Morgan C, Adde L, Blackman J, Boyd RN, Brunstrom-Hernandez J, et al. Early, Accurate Diagnosis and Early Intervention in Cerebral Palsy: Advances in Diagnosis and Treatment. JAMA Pediatr. 2017 Sep 1;171(9):897–907.

12. King AR, Al Imam MH, McIntyre S, Morgan C, Khandaker G, Badawi N, et al. Early Diagnosis of Cerebral Palsy in Low- and Middle-Income Countries. Brain Sci. 2022 Apr 23;12(5):539.

13. Tomantschger I, Herrero D, Einspieler C, Hamamura C, Voos MC, Marschik PB. The general movement assessment in non-European low- and middle-income countries. Rev Saude Publica. 2018 Jan 29;52(6):1–8.

14. Fleming KA, Horton S, Wilson ML, Atun R, DeStigter K, Flanigan J, et al. The Lancet Commission on diagnostics: transforming access to diagnostics. The Lancet. 2021;398(10315):1997–2050.

15. World Health Organization. WHO guideline recommendations on digital interventions for health system strengthening. 2019;

For peer review only

For peer review only - http://bmjopen.bmj.com/site/about/guidelines.xhtml

16. Spittle A, Olsen J, Kwong A, Doyle L, Marschik P, Einspieler C, et al. The Baby Moves prospective cohort study protocol: using a smartphone application with the General Movements Assessment to predict neurodevelopmental outcomes at age 2 years for extremely preterm or extremely low birthweight infants. BMJ open. 2016;6(10):e013446.

17. Adde L, Brown A, Van Den Broeck C, Decoen K, Eriksen BH, Fjørtoft T, et al. In-Motion-App for remote General Movement Assessment: A multi-site observational study. BMJ Open. 2021;11(3).

18. Svensson KA, Örtqvist M, Bos AF, Eliasson AC, Sundelin HEK. Usability and inter-rater reliability of the NeuroMotion app: A tool in General Movements Assessments. European Journal of Paediatric Neurology. 2021 Jul;33:29–35.

19. Silva N, Zhang D, Kulvicius T, Gail A, Barreiros C, Lindstaedt S, et al. The future of General Movement Assessment: The role of computer vision and machine learning - A scoping review. Res Dev Disabil. 2021 Mar;110:103854.

20. Vandenbroucke JP, Elm E von, Altman DG, Gøtzsche PC, Mulrow CD, Pocock SJ, et al. Strengthening the Reporting of Observational Studies in Epidemiology (STROBE): Explanation and Elaboration. PLOS Medicine. 2007 Oct 16;4(10):e297.

21. Eldridge SM, Chan CL, Campbell MJ, Bond CM, Hopewell S, Thabane L, et al. CONSORT 2010 statement: extension to randomised pilot and feasibility trials. Pilot Feasibility Stud. 2016;2:64.

22. Lancaster GA, Thabane L. Guidelines for reporting non-randomised pilot and feasibility studies. Pilot and Feasibility Studies. 2019 Oct 6;5(1):114.

23. Ministry of Health and Population, Nepal, New ERA, Kathmandu, ICF. Nepal Demographic and Health Survey 2022: Key Indicators Report. Ministry of Health and Population, Kathmandu, Nepal; 2022. ‘

24. Thapa R. Symptom Recognition and Diagnosis of Cerebral Palsy in Nepal. J Autism Dev Disord. 2017;47(6):1739–48.

25. Banskota B, Shrestha S, Rajbhandari T, Banskota AK, Spiegel DA. A Snapshot of 1001 Children Presenting with Cerebral Palsy to a Children’s Disability Hospital. J Nepal Health Res Counc. 2015 Apr;13(29):31–7.

26. World Health Organization Regional Office for South-East Asia. Compilation of community-based rehabilitation practices in the WHO South-East Asia Region; 2013

27. Buderer NM. Statistical methodology: I. Incorporating the prevalence of disease into the sample size calculation for sensitivity and specificity. Acad Emerg Med Off J Soc Acad Emerg Med. 1996 Sep;3(9):895–900.

For peer review only

28. Kc A, Wrammert J, Clark RB, Ewald U, Vitrakoti R, Chaudhary P, et al. Reducing Perinatal Mortality in Nepal Using Helping Babies Breathe. Pediatrics. 2016;137(6).

29. Kwong AK, Eeles AL, Olsen JE, Cheong JL, Doyle LW, Spittle AJ. The Baby Moves smartphone app for General Movements Assessment: Engagement amongst extremely preterm and term-born infants in a state-wide geographical study. J Paediatr Child Health. 2018 Oct 4;

30. Proctor E, Silmere H, Raghavan R, Hovmand P, Aarons G, Bunger A, et al. Outcomes for implementation research: Conceptual distinctions, measurement challenges, and research agenda. Administration and Policy in Mental Health and Mental Health Services Research. 2011;38(2):65–76.

31. Hayes A, Krippendorff K. Answering the Call for a Standard Reliability Measure for Coding Data. Communication Methods and Measures. 2007 Apr 1;1:77–89.

32. Kwong AKL, Eeles AL, Olsen JE, Zannino D, Kariotis T, Spittle AJ. Instructional guides for filming infant movements at home are effective for the General Movements Assessment. Journal of Paediatrics and Child Health. 2021;58(5):796–801.

33. Saini L, Madaan P, Bhagwat C, Einspieler C. Home-videos for neurodevelopmental follow-up of high-risk infants during COVID-19 pandemic: a simple and inexpensive tool. Journal of Tropical Pediatrics. 2021;67(1):fmaa088.

34. United Nations General Assembly. Transforming our world: the 2030 Agenda for Sustainable Development. Resolution 70/1. 2015.

35. Caesar R, Colditz PB, Cioni G, Boyd RN. Clinical tools used in young infants born very preterm to predict motor and cognitive delay (not cerebral palsy): a systematic review. Dev Med Child Neurol. 2021 Apr;63(4):387–95.

36. Maitre NL, Benninger KL, Neel ML, Haase JA, Pietruszewski L, Levengood K, et al. Standardized Neurodevelopmental Surveillance of High-risk Infants Using Telehealth: Implementation Study during COVID-19. Pediatr Qual Saf. 2021 Jul 28;6(4):e439.

37. Saul R, Phillips D. Ghosts and germs: Cerebral Palsy in Nepal: A preliminary exploration of cosmology and disability. Disability and development: Learning from action and research on disability in the majority world. 1999;

38. Byrne R, Duncan A, Pickar T, Burkhardt S, Boyd RN, Neel ML, et al. Comparing parent and provider priorities in discussions of early detection and intervention for infants with and at risk of cerebral palsy. Child: Care, Health and Development. 2019;45(6):799–807.

For peer review only - http://bmjopen.bmj.com/site/about/guidelines.xhtml

Figure and Table Legends

Figure 1: Screenshots of the Nepali version of the NeuroMotion™ app including a) introduction, b-d) instructions about filming, and e) a view of the filming interface. Corresponding sections in the English version read: a) This application is designed in order to record and send short clips in a simple and secure way from a smartphone to a secure server only available to healthcare personnel. The clips can be used as support in medical research and diagnostics. b) Hello! With this app you can record your child. The film can be uploaded and analysed by healthcare personnel. c) Remember! Keep the camera as still as possible, same distance and direction. Head, hands and feet visible in the screen. Background as neutral as possible. d) A baby: Should be on its back, be active and satisfied. Only wear one-coloured t-shirt / onepiece and diaper, no pacifier. Film from above, so that the whole child can be seen on the screen. The recording should be t minutes.

Figure 2: Participant flow diagram. FM =Fidgety Movements, SNCU/NICU = Sick Newborn Care Unit/Neonatal Intensive Care Unit

Figure 3: Stacked bar graph of parental answers to smartphone app usability survey.

Table 1: Background characteristics. Comparison between those participants who returned at least one film of approved technical quality and those who did not.

For peer review only - http://bmjopen.bmj.com/site/about/guidelines.xhtml

For peer review only

For peer review only - http://bmjopen.bmj.com/site/about/guidelines.xhtml

Supporting information

Supplementary Table 1: General Movements trust specialist assessment of the technical quality of the NeuroMotion™ films.

Supplementary Table 2: Results of the technical quality assessment and evaluation of Fidgety Movements.

For peer review only

For peer review only - http://bmjopen.bmj.com/site/about/guidelines.xhtml

Table 1:

	Filmed successfully = 16	Not filmed successfully = 15	p-value	cOR (95%CI)
INFANT CHARACTERISTICS				
Gestational age in weeks, Mean (SD)	38.9 (1.4)	38.8 (1.5)	0.32*	-
Apgar at 5 min, Mean (SD)	5.3 (0.4)	4.8 (0.7)	0.67*	-
Resuscitation at 5 min	16	14	0.29**	NA
Clinical seizures	3	7	0.14***	0.26 (0.05-1.32)
Sex			1.00***	
Female	3	3		Ref
Male	13	12		1.08 (0.18-6.42)
Mode of delivery			0.25***	
Normal vaginal	13	9		Ref
Instrumental	0	0		-
Caesarean section	3	6		0.34 (0.06-1.75)
DEMOGRAPHIC FACTORS				
Maternal education			0.36***	
1-7 years	5	3		Ref
None	0	2		-
Complete primary	3	2		0.45 (0.05-3.57)
Complete secondary	3	6		0.30 (0.04-2.20-
University degree	5	2		1.50 (0.17-13.22)
Maternal ethnicity			0.90**	
Relatively disadvantageous	11	10		Ref
Relatively advantageous	5	5		1.10 (0.24-4.96)
Maternal age, Mean (SD)	25y5m (3y6m)	24y2m (3y)	0.60*	
Paternal ethnicity			0.90**	
Relatively disadvantageous	11	10		Ref
Relatively advantageous	5	5		1.10 (0.24-4.96)
Paternal age, Mean (SD)	29y6m (4y7m)	27y7m (3y3m)	0.37*	

For peer review only - http://bmjopen.bmj.com/site/about/guidelines.xhtml

	Filmed successfully = 16	Not filmed successfully = 15	p-value	cOR (95%CI)
SMARTPHONE USE				
Parents own a smart phone	16	15	NA	-
Years with smart phone, Mean (SD)	9y1m (3y2m)	7y7m (3y)	0.68*	-
Parents use social media	15	15	0.33**	NA
App installation			1.00***	
Hospital	5	4	1.00	Ref
Home	11	11	1.00	0.80 (0.16-3.79)

CI = Confidence Interval, cOR = crude Odds Ratio, SD = Standard Deviation. No missing data. *independent samples t-test, ** Pearson's chi-square test if a cell has count 5 or more, ***Fischer's exact text if a cell has count less than 5. Parental ethnicity was self-reported. Relatively disadvantageous = Dalit, Janajati, Madhesi or Muslim, Relatively advantageous = Brahmini, Chhetri or other.

For peer review only - http://bmjopen.bmj.com/site/about/guidelines.xhtml

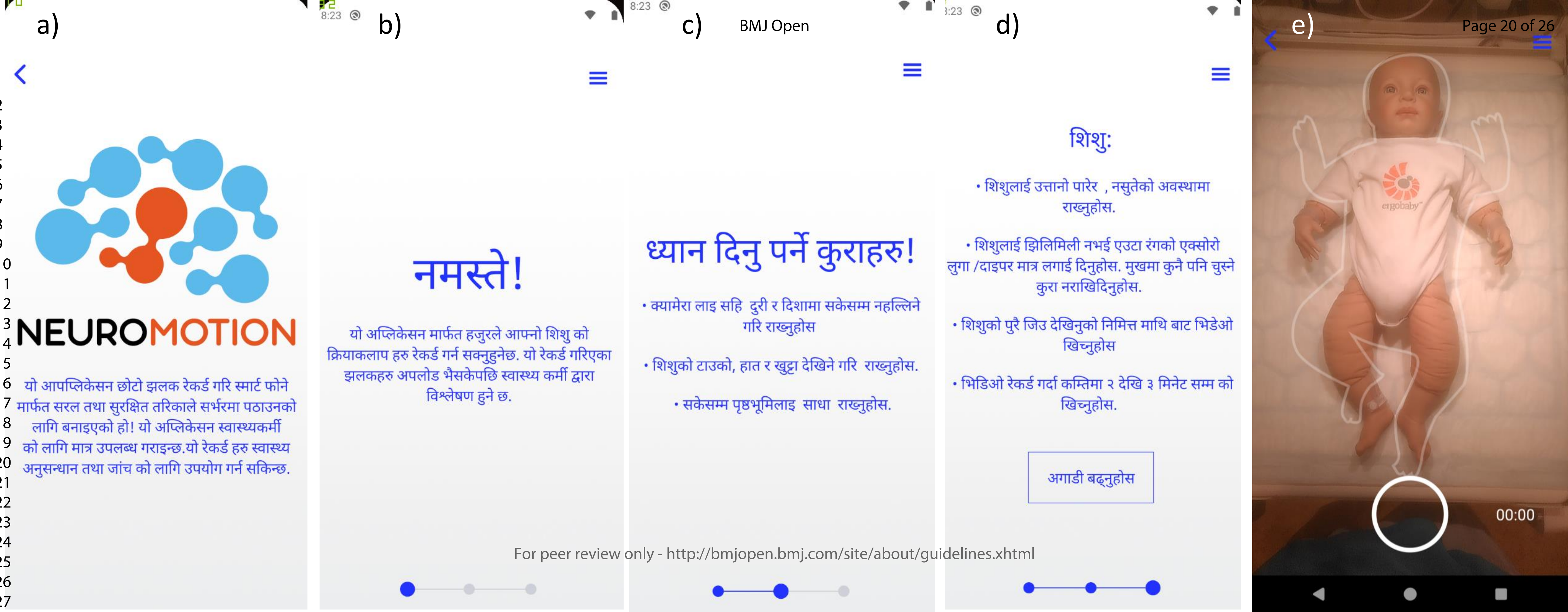

For peer review only - http://bmjopen.bmj.com/site/about/guidelines.xhtml

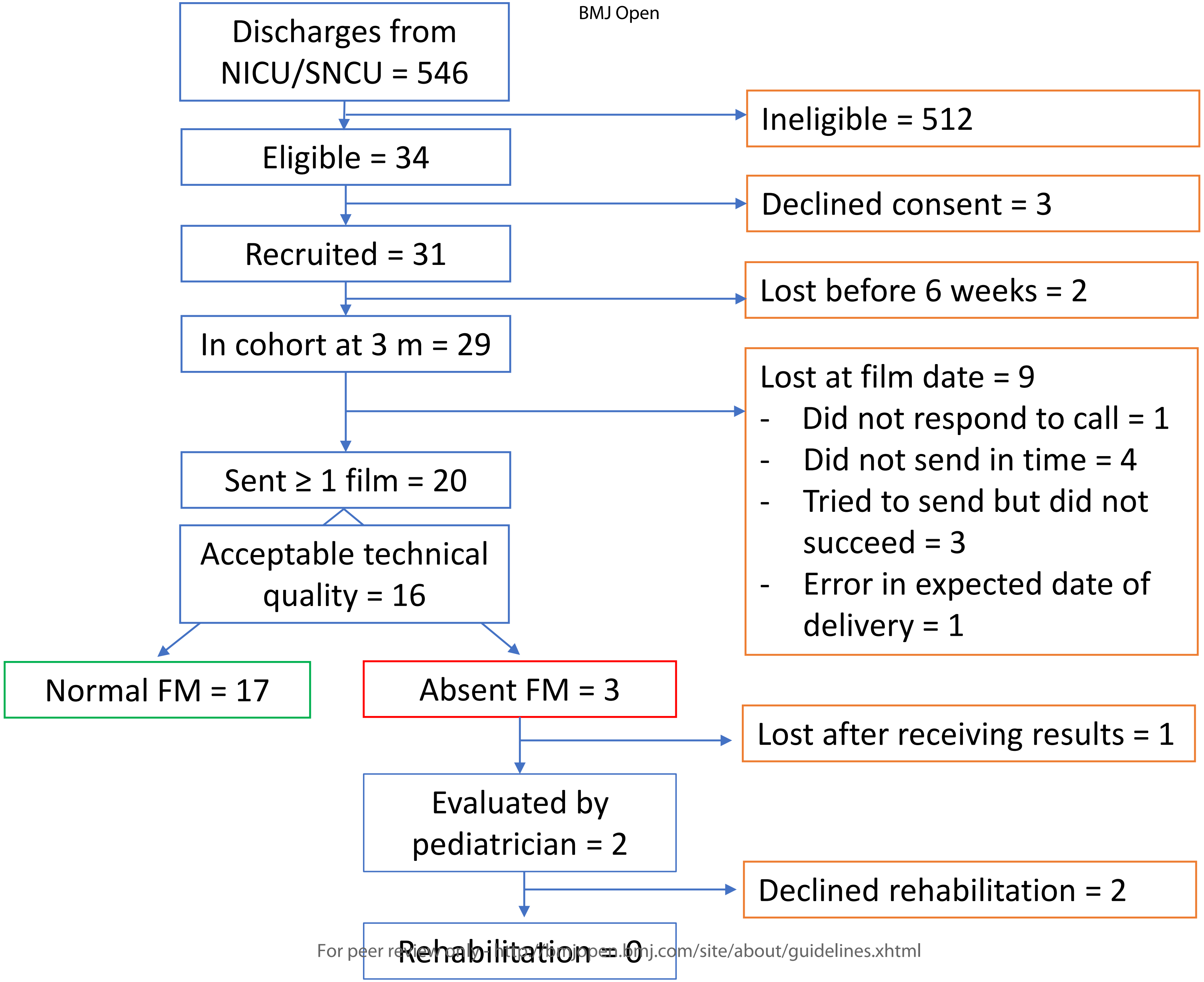
Discharges from NICU/SNCU = 546
Ineligible = 512
Eligible = 34
Declined consent = 3
Recruited = 31
Lost before 6 weeks = 2
In cohort at 3 m = 29
Lost at film date = 9
- Did not respond to call = 1
- Did not send in time = 4
- Tried to send but did not succeed = 3
- Error in expected date of delivery = 1
Sent ≥ 1 film = 20
Acceptable technical quality = 16
Normal FM = 17
Absent FM = 3
Lost after receiving results = 1
Evaluated by pediatrician = 2
Declined rehabilitation = 2
Rehabilitation = 0

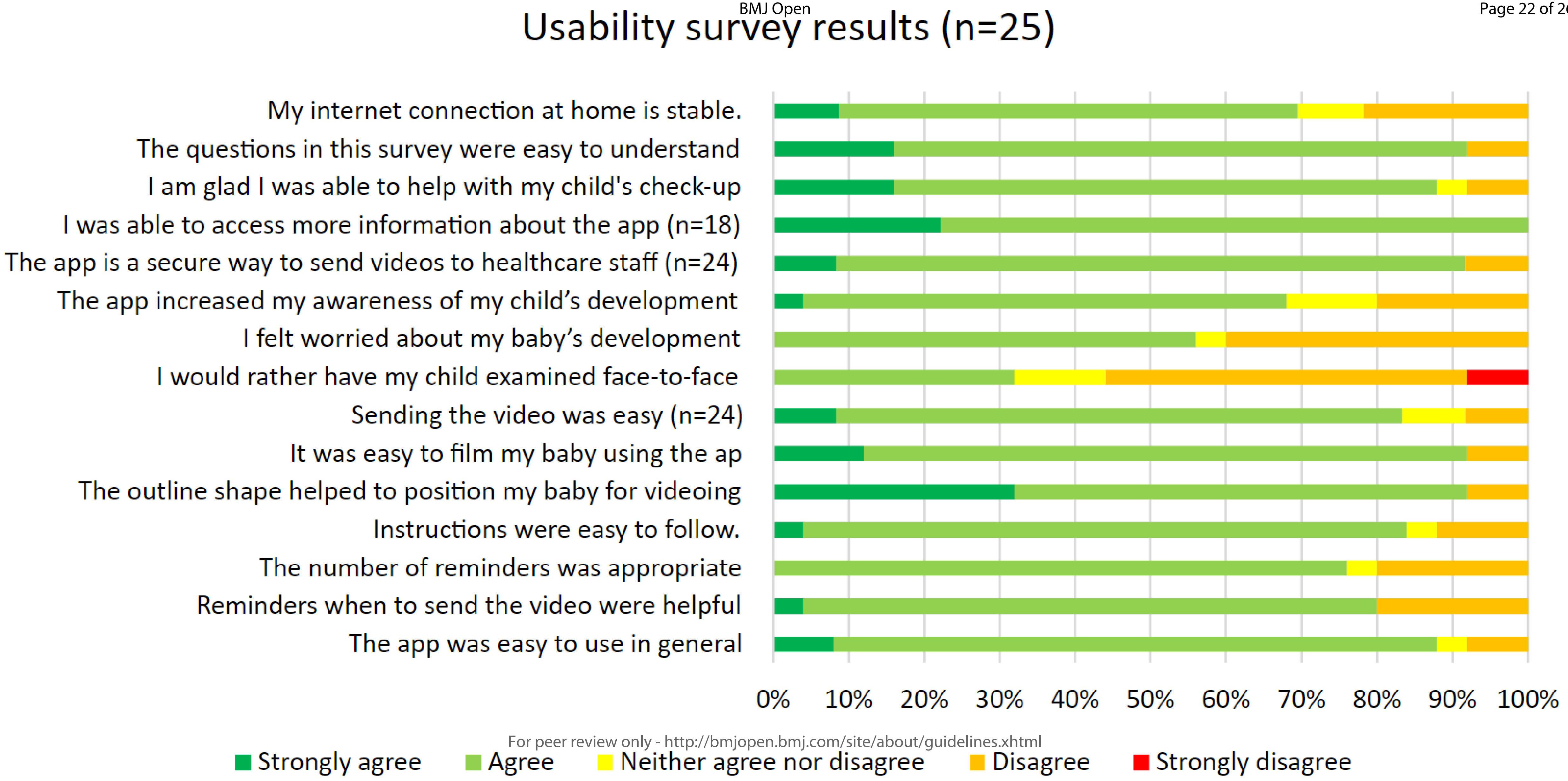
Usability survey results (n=25)
My internet connection at home is stable.
The questions in this survey were easy to understand
I am glad I was able to help with my child's check-up
I was able to access more information about the app (n=18)
The app is a secure way to send videos to healthcare staff (n=24)
The app increased my awareness of my child's development
I felt worried about my baby's development
I would rather have my child examined face-to-face
Sending the video was easy (n=24)
It was easy to film my baby using the ap
The outline shape helped to position my baby for videoing
Instructions were easy to follow.
The number of reminders was appropriate
Reminders when to send the video were helpful
The app was easy to use in general
0%
10%
20%
30%
40%
50%
60%
70%
80%
90%
100%
Strongly agree
Agree
Neither agree nor disagree
Disagree
Strongly disagree

Film #

Assessed by:____

Date:____

Quality	Approved		Not approved	Comments
	Excellent	Good	Indistinct	
Picture quality				
Sharpness				
Camera is held in correct position				
Camera is held in a steady position				
Baby setting				
Clothing				
Background/blanket				
Supine position				
Surrounding influences				
Baby´s mood				
Overall judgement:				

For peer review only

		Picture quality			Baby setting					Overall judgement	Fidgety Movements
Film number	Case number	Sharpness	Camera held in correct pos.	Camera held steady	Clothing	Background/ blanket	Supine position	Surrounding influences	Baby's mood		
1	1	G	E	G	E	E	E	E	E	Approved	F-
2	1	G	I	G	E	E	E	E	E	Approved	F-
3	1	G	G	G	E	E	E	E	E	Approved	F-
4	2	E	I	I	E	G	E	E	E	Approved	F+
5	2	E	I	I	E	E	E	E	E	Rejected	?
6	2	E	I	I	E	G	E	E	E	Approved	F+
7	3	E	E	I	E	G	E	E	E	Approved	F+
8	4	E	E	G	E	G	E	E	E	Approved	F+
9	4	E	E	G	E	G	E	E	E	Approved	F+
10	4	E	E	G	E	G	E	G	E	Approved	F+
11	4	E	E	G	E	G	G	I	E	Rejected	F+
12	4	E	I	I	E	G	G	I	E	Rejected	?
13	4	E	E	E	E	G	E	G	E	Approved	F+
14	5	E	E	E	G	E	E	E	E	Approved	F+
15	5	E	E	E	G	I	E	E	E	Approved	F+
16	5	E	G	E	E	G	E	E	E	Approved	F+
17	5	E	E	E	E	E	E	E	E	Approved	F+
18	5	E	E	E	E	E	E	E	E	Approved	F+
19	6	E	E	I	E	E	E	E	E	Approved	F+
20	6	E	E	G	E	E	E	E	E	Approved	F+
21	7	E	E	G	E	I	E	I	G	Approved	F+
22	7	E	E	I	G	I	E	E	E	Approved	F+
23	8	E	I	I	G	G	E	I	E	Rejected	F+
24	9	G	G	G	E	I	E	G	E	Approved	F+
25	10	E	G	G	E	G	E	G	E	Approved	F-
26	10	G	E	I	E	I	E	I	E	Approved	F-
27	10	E	G	I	E	G	E	G	E	Approved	F-
28	11	E	I	I	I	I	E	E	E	Rejected	?
29	11	E	I	I	E	E	E	I	E	Rejected	F+
30	12	I	E	G	I	G	E	E	E	Rejected	?
31	12	I	G	G	I	I	G	E	E	Rejected	F+
32	12	E	G	E	G	E	E	E	E	Approved	F+
33	12	E	G	G	I	I	E	I	E	Approved	F+
34	13	E	G	G	E	E	E	E	E	Approved	F+
35	13	E	E	G	E	E	E	E	E	Approved	F+
36	14	E	I	I	E	I	E	I	E	Rejected	F-(?)
37	15	E	G	G	E	E	E	E	E	Approved	F+

For peer review only

38	16	E	I	I	I	I	E	I	E	Rejected	?
39	16	E	E	E	G	E	E	E	E	Approved	F+
40	16	E	E	E	G	E	E	E	E	Approved	F+
41	17	E	E	E	E	E	E	E	E	Approved	F+
42	18	E	E	E	E	E	E	E	E	Approved	F+
43	18	E	E	E	E	E	E	G	E	Approved	F+
44	19	E	G	E	E	E	E	E	E	Approved	F+
45	19	E	I	G	E	G	E	E	E	Approved	F+
46	20	E	I	I	E	I	E	E	E	Rejected	F+
Summary of results	Excellent	39	23	13	34	20	43	31	45		
	Good	5	11	18	7	15	3	6	1		
	Indistinct	2	12	15	5	11	0	9	0		
Films number 11, 23, 29 and 31 were rejected for technical quality, but Fidgety Movements were clearly observed.											
Case number 14 only sent one film that was rejected due to poor technical quality, but Fidgety Movements were not observed.											

For peer review only

STROBE Statement—Checklist of items that should be included in reports of ***cohort studies***

	Item No	Recommendation	
**Title and abstract**	1	(*a*) Indicate the study's design with a commonly used term in the title or the abstract	1
		(*b*) Provide in the abstract an informative and balanced summary of what was done and what was found	2
**Introduction**			
Background/rationale	2	Explain the scientific background and rationale for the investigation being reported	3
Objectives	3	State specific objectives, including any prespecified hypotheses	5
**Methods**			
Study design	4	Present key elements of study design early in the paper	3
Setting	5	Describe the setting, locations, and relevant dates, including periods of recruitment, exposure, follow-up, and data collection	3-5
Participants	6	(*a*) Give the eligibility criteria, and the sources and methods of selection of participants. Describe methods of follow-up	4
		(*b*) For matched studies, give matching criteria and number of exposed and unexposed	N/A
Variables	7	Clearly define all outcomes, exposures, predictors, potential confounders, and effect modifiers. Give diagnostic criteria, if applicable	5
Data sources/ measurement	8*	For each variable of interest, give sources of data and details of methods of assessment (measurement). Describe comparability of assessment methods if there is more than one group	4,5
Bias	9	Describe any efforts to address potential sources of bias	8
Study size	10	Explain how the study size was arrived at	4
Quantitative variables	11	Explain how quantitative variables were handled in the analyses. If applicable, describe which groupings were chosen and why	5, table 1
Statistical methods	12	(*a*) Describe all statistical methods, including those used to control for confounding	5
		(*b*) Describe any methods used to examine subgroups and interactions	N/A
		(*c*) Explain how missing data were addressed	N/A
		(*d*) If applicable, explain how loss to follow-up was addressed	not relevant
		(*e*) Describe any sensitivity analyses	N/A
**Results**			
Participants	13*	(a) Report numbers of individuals at each stage of study—eg numbers potentially eligible, examined for eligibility, confirmed eligible, included in the study, completing follow-up, and analysed	Fig 1
		(b) Give reasons for non-participation at each stage	Fig 1
		(c) Consider use of a flow diagram	Fig 1
Descriptive data	14*	(a) Give characteristics of study participants (eg demographic, clinical, social) and information on exposures and potential confounders	Table 1
		(b) Indicate number of participants with missing data for each variable of interest	table 1
		(c) Summarise follow-up time (eg, average and total amount) N/A	ingress
Outcome data	15*	Report numbers of outcome events or summary measures over time	
Main results	16	(*a*) Give unadjusted estimates and, if applicable, confounder-adjusted estimates and their precision (eg, 95% confidence interval). Make clear which confounders were adjusted for and why they were included only cOR reported	
		(*b*) Report category boundaries when continuous variables were categorized	not relevant
		(*c*) If relevant, consider translating estimates of relative risk into absolute risk for a meaningful time period	

For peer review only

Other analyses	17	Report other analyses done—eg analyses of subgroups and interactions, and sensitivity analyses	N/A
**Discussion**			
Key results	18	Summarise key results with reference to study objectives	7
Limitations	19	Discuss limitations of the study, taking into account sources of potential bias or imprecision. Discuss both direction and magnitude of any potential bias	8
Interpretation	20	Give a cautious overall interpretation of results considering objectives, limitations, multiplicity of analyses, results from similar studies, and other relevant evidence	7
Generalisability	21	Discuss the generalisability (external validity) of the study results	8
**Other information**			
Funding	22	Give the source of funding and the role of the funders for the present study and, if applicable, for the original study on which the present article is based	12

*Give information separately for exposed and unexposed groups.

Note: An Explanation and Elaboration article discusses each checklist item and gives methodological background and published examples of transparent reporting. The STROBE checklist is best used in conjunction with this article (freely available on the Web sites of PLoS Medicine at http://www.plosmedicine.org/, Annals of Internal Medicine at http://www.annals.org/, and Epidemiology at http://www.epidem.com/). Information on the STROBE Initiative is available at http://www.strobe-statement.org.

For peer review only
